# Changes in levels of the antioxidant glutathione in brain and blood across the age span of healthy adults: A systematic review

**DOI:** 10.1016/j.nicl.2023.103503

**Published:** 2023-08-26

**Authors:** Flavie Detcheverry, Sneha Senthil, Sridar Narayanan, AmanPreet Badhwar

**Affiliations:** a Multiomics Investigation of Neurodegenerative Diseases (MIND) lab, Montreal, QC, Canada; b Département de Pharmacologie et Physiologie, Faculté de Médecine, Université de Montréal, Montreal, QC, Canada; c Institut de Génie Biomédical, Université de Montréal, Montreal, QC, Canada; d Centre de Recherche de l’Institut Universitaire de Gériatrie de Montréal (CRIUGM), Montreal, QC, Canada; e Department of Neurology and Neurosurgery, Faculty of Medicine, McGill University, Montreal, QC, Canada; f McConnell Brain Imaging Centre, Montreal Neurological Institute-Hospital, Montreal, QC, Canada

**Keywords:** Antioxidant, Glutathione, Healthy aging, Magnetic resonance spectroscopy, Biochemical assays, Oxidative stress

## Abstract

Aging is characterized by a gradual decline of the body’s biological functions, which can lead to increased production of reactive oxygen species (ROS). Antioxidants neutralize ROS and maintain balance between oxidation and reduction. If ROS production exceeds the ability of antioxidant systems to neutralize, a damaging state of oxidative stress (OS) may exist. The reduced form of glutathione (GSH) is the most abundant antioxidant, and decline of GSH is considered a marker of OS. Our review summarizes the literature on GSH variations with age in healthy adults in brain (*in vivo*, *ex vivo*) and blood (plasma, serum), and reliability of *in vivo* magnetic resonance spectroscopy (MRS) measurement of GSH. A systematic PubMed search identified 35 studies. All *in vivo* MRS studies (N = 13) reported good to excellent reproducibility of GSH measures. In brain, 3 out of 4 MRS studies reported decreased GSH with age, measured in precuneus, cingulate, and occipital regions, while 1 study reported increased GSH with age in frontal and sensorimotor regions. In post-mortem brain, out of 3 studies, 2 reported decreased GSH with age in hippocampal and frontal regions, while 1 study reported increased GSH with age in a frontal region. Oxidized glutathione disulfide (GSSG) was reported to be increased in caudate with age in 1 study, suggesting OS. Although findings in the brain lacked a clear consensus, the majority of studies suggested a decline of GSH with age. The low number of studies (particularly *ex vivo*) and potential regional differences may have contributed to variability in the findings in brain. In blood, in contrast, GSH levels predominately were reported to decrease with advancing age (except in the oldest-old, who may represent a select group of particularly successful agers), while GSSG findings lacked consensus. The larger number of studies assessing age-specific GSH level changes in blood (N = 16) allowed for more robust consensus across studies than in brain. Overall, the literature suggests that aging is associated with increased OS in brain and body, but the timing and regional distribution of changes in the brain require further study. The contribution of brain OS to brain aging, and the effect of interventions to raise brain GSH levels on decline of brain function, remain understudied. Given that reliable tools to measure brain GSH exist, we hope this paper will serve as a catalyst to stimulate more work in this field.

## Highlights

• Excess reactive oxygen species in aging can cause oxidative stress and cell damage.
• Glutathione (GSH) neutralizes reactive oxygen species and combats oxidative stress.
• We systematically reviewed GSH variations in brain and blood in healthy aging.
• In brain, region-specific GSH differences have been reported with increasing age.
• In blood, GSH levels generally decline with age across studies.

## Graphical abstract

**Figure:**
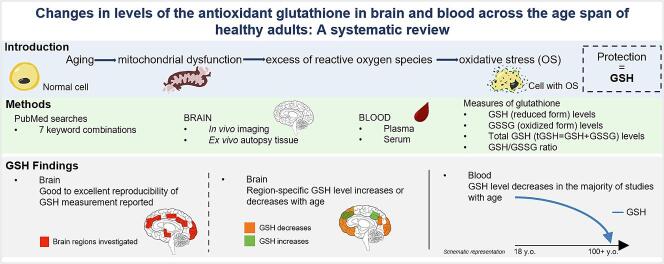

## 1 Introduction

**Fig. 1:**
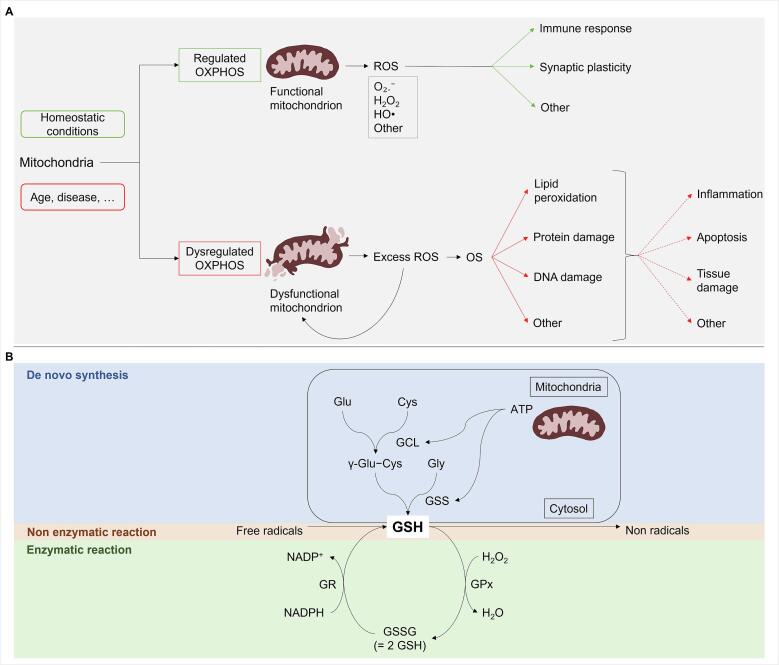
Mitochondrial OXPHOS, ROS and the antioxidant glutathione. A) Relationship between the aging process, mitochondrial OXPHOS, ROS production and OS; and B) GSH-facilitated ROS quenching. Note: two GSH on the sulfur atom compose GSSG (Pizzorno, 2014), hence the formula GSSG = 2 GSH. Abbreviation: ATP, adenosine triphosphate; Cys, cysteine; DNA, deoxyribonucleic acid; γ-Glu-Cys, gamma-L-Glutamyl-L-cysteine; GCL, glutamate-cysteine ligase; Glu, glutamic acid; Gly, glycine; GPx, glutathione peroxidase; GR, glutathione reductase; GSH, glutathione; GSS, glutathione synthetase; GSSG, glutathione disulfide; HO•, hydroxyl radical; H_2_O, water; H_2_O_2_, hydrogen peroxide; NADPH, nicotinamide adenine dinucleotide phosphate; NADP+, nicotinamide adenine dinucleotide phosphate, accepts electrons to form NADPH; OS, oxidative stress; O₂.^-^, superoxide; OXPHOS, oxidative phosphorylation; ROS, reactive oxygen species.

The population worldwide is aging, and it is estimated that over 2.1 billion individuals (22% of the world's population) will be 60 years (yrs) or older by the year 2050 (Ageing and Health, 2022). Aging is characterized by a gradual decline of the body’s biological functions, including metabolic homeostasis (MacDonald and Pike, 2021). Essential for maintaining the efficiency of life-sustaining chemical reactions, metabolic homeostasis relies on a balance between anabolic and catabolic pathways, the latter being critical for energy production (Pflug et al., 2021). Playing a central role in energy production is the mitochondrion, a double membrane bound cellular organelle (Cheng and Ristow, 2013). During energy production via the catabolic oxidative phosphorylation (OXPHOS) pathway, mitochondria produce reactive oxygen species (ROS) that are highly reactive chemicals formed from diatomic oxygen (O_2_). Examples of ROS include superoxide anion radical (O₂.^-^), hydrogen peroxide (H_2_O_2_), and hydroxyl radical (HO•) (Ray et al., 2012). As demonstrated in Fig. 1A, under homeostatic conditions, ROS play a beneficial role in biological processes, such as immune response and synaptic plasticity (Cheng and Ristow, 2013, Grimm and Eckert, 2017). However, excess ROS production can lead to oxidative stress (OS) (Grimm and Eckert, 2017), which causes damage to lipids, proteins and deoxyribonucleic acid (DNA), leading eventually to inflammation, apoptotic cell death, and tissue damage (Fig. 1A) (Pareek et al., 2019).

The free radical theory of aging posits that excess ROS-induced damage significantly contributes to aging and age-associated diseases (Grimm and Eckert, 2017, Wickens, 2001). With the highest energy needs of all organs in the body, the brain is extremely vulnerable to mitochondrial dysfunction and associated ROS overproduction and OS (Grimm and Eckert, 2017). To protect against the harmful effects of OS and maintain metabolic homeostasis, the brain (like the rest of the body) uses antioxidants such as glutathione, superoxide dismutase, catalase, and vitamins C and E to neutralize excess ROS (Dwivedi et al., 2020). Of these, glutathione is the most prevalent endogenous antioxidant in the body, including the brain (Bottino et al., 2021, Pocernich and Butterfield, 2012). In the body, glutathione exists in both thiol-reduced (GSH) and disulfide-oxidized (glutathione disulfide; GSSG) forms, with GSH being the predominant form (Lu, 2013). Total glutathione (tGSH) is thus the sum of GSH and GSSG.

As illustrated in Fig. 1B, GSH is synthesized in the cytosol by two adenosine triphosphate (ATP)-dependent steps. The first step is the combination of amino acids glutamate and cysteine by γ-glutamyl cysteine synthetase, also known as glutamate-cysteine ligase (GCL), to form gamma-glutamate-cysteine (γ-Glu-Cys) (Bottino et al., 2021, Lu, 2013, Wu et al., 2004). GCL is a heterodimeric protein formed of two subunits, namely GCL modifier subunit (GCLM) and GCL catalytic subunit (GCLC), which are encoded by different genes (Lu, 2013). In the second step, the dipeptide γ-Glu-Cys is combined with the amino acid glycine by glutathione synthetase (GSS) to form the tripeptide GSH (Bottino et al., 2021, Lu, 2013, Wu et al., 2004). As an antioxidant, GSH plays a fundamental role in regulating the intracellular redox environment, detoxification/quenching of ROS, and supporting oxidative defense (Saharan and Mandal, 2014). GSH can neutralize/reduce ROS both directly and enzymatically (Fig. 1B). During the latter, GSH acts as a reducing agent and donates an electron in the presence of the enzyme glutathione peroxidase (GPx), thereby giving rise to its oxidized form, GSSG (Higuchi, 2014). Converting GSSG back to GSH is carried out by the enzyme glutathione reductase (GR) (Tong et al., 2016). In general, significant decreases in GSH levels (Ballatori et al., 2009) or the GSH/GSSG ratio (Sadhu et al., 2016, Zitka et al., 2012) are indicative of OS.

With increasing age, GSH levels are known to be altered in the brain in both humans (Emir et al., 2011) and other animals (Zhu et al., 2006). For example in older mice, damage in the antioxidant system occurs, and is associated with lower mitochondrial GSH (Shi et al., 2010) and superoxide dismutase activities in the neocortex (Wang et al., 2015), as well as decreased GSH/GSSG ratio in the cortex, striatum, hippocampus and cerebellum (Rebrin et al., 2007). In humans, GSH levels can be assessed in the brain *in vivo* using magnetic resonance spectroscopy (MRS), a powerful non-invasive technique for cerebral metabolite quantification, as well as in autopsy brain tissue and peripheral blood using biochemical assays, such as high-performance liquid chromatography (HPLC) (Asensi et al., 1994). While literature suggests GSH levels change as a function of age, the consistency of these alterations across studies have not been assessed. We address this knowledge gap by conducting a systematic review of the literature to examine consistency of age-specific GSH level changes in brain and peripheral blood (plasma, serum), considered a systemic (i.e., whole body including the brain) readout.

## 2 Methods

### 2.1 Search strategy and study selection

A systematic search was conducted up to November 2022 in accordance with the Preferred Reporting Items for Systematic Reviews and Meta-Analyses guidelines (PRISMA), to identify original studies from the PubMed database (PubMed, 2006) investigating GSH levels in (i) brain – *in vivo,* using proton MRS (^1^H-MRS) of healthy adults, or *ex vivo* biochemical analyses of autopsy tissue from people without neurological or psychiatric conditions in life, and/or (ii) blood from healthy adults – using biochemical analyses of the fluid component (i.e., plasma or serum). Using the operators “AND” and “OR”, and the filters “Humans” and “English”, we conducted searches using the following seven expressions: Search 1: (“MRS” OR “magnetic resonance spectroscopy”) AND (“GSH” OR “glutathione”) AND “reproducibility”; Search 2: (“MRS” OR “magnetic resonance spectroscopy”) AND (“GSH” OR “glutathione”) AND “aging”; Search 3: (“brain tissue” OR “brain autopsy”) AND (“GSH” OR “glutathione”) AND “aging”; Search 4: “plasma” AND (“GSH” OR “glutathione”) AND “aging”; Search 5: “serum” AND (“GSH” OR “glutathione”) AND “aging”; Search 6: “brain” AND (“GSH” OR “glutathione”) AND “aging”; and Search 7: “blood” AND (“GSH” OR “glutathione”) AND “aging”. Search results were filtered for duplicates, and unique studies investigating age-related changes in GSH levels in adult humans were included. In general, we considered individuals 18 to 39 yrs of age as young adults, 40 to 59 yrs of age as middle-aged adults, and ≥60 yrs of age as older adults (The Editors of Encyclopedia Britannica, 2018). Also included were papers investigating tGSH, GSSG and the GSH/GSSG ratio.

Studies meeting the following exclusion criteria were excluded: non glutathione studies; no healthy adults or healthy age comparison; studies not investigating glutathione levels in brain or blood-plasma or -serum; animal or cell culture studies; human adults with neurological or psychiatric conditions only; studies not giving sufficient information about age; MRS studies using a different nucleus than proton (i.e., phosphorus-31 or carbon-13); case control studies; clinical trial or intervention without baseline data; review articles or meta analysis; and studies not published in English. In addition, for the purposes of this review, we focused on GSH level readout in the liquid portion of blood (plasma or serum), since we were interested in the systemic readout of GSH. We, therefore, excluded studies investigating GSH levels in erythrocytes, as this readout would be specific to the blood tissue itself.

### 2.2 Data extraction

Two reviewers (F.D. and A.B.) conducted the searches and one (F.D.) checked for duplicates. Two reviewers (F.D. and A.B.) independently screened all unique search results for potential inclusion in the review. Data extracted from studies that met inclusion criteria were as follows: number of study participants, age range of participants, sex distribution, method(s) used to assess glutathione (GSH (reduced form), GSSG (oxidized form), tGSH, or GSH/GSSG ratio), and variations in metabolite levels with age. Specific to the brain studies (both *in vivo* and in autopsy tissue) the region of interest (ROI) was noted. For *in vivo* brain studies using MRS, magnetic field strength and the pulse sequence used were noted. For MRS studies assessing the reproducibility of GSH quantification, we also noted the echo time (TE), metabolite referencing scheme (i.e., water reference, absolute quantitation, ratio to creatine), and reported reproducibility statistics. GSH results from studies using the same cohort were pooled under the PubMed unique identifier or PMID of the earliest publication and treated as results from a single study to avoid counting the cohort multiple times. Moreover, the risk of bias for included studies was assessed by two independent reviewers (F.D. and S.S.) using the Joanna Briggs Institute (JBI)’s analytical Cross Sectional studies guidelines (Haile, 2022). Inter-rater conflicts were resolved via discussion and a re-review of the article.

## 3 Results

### 3.1 Search results

**Fig. 2:**
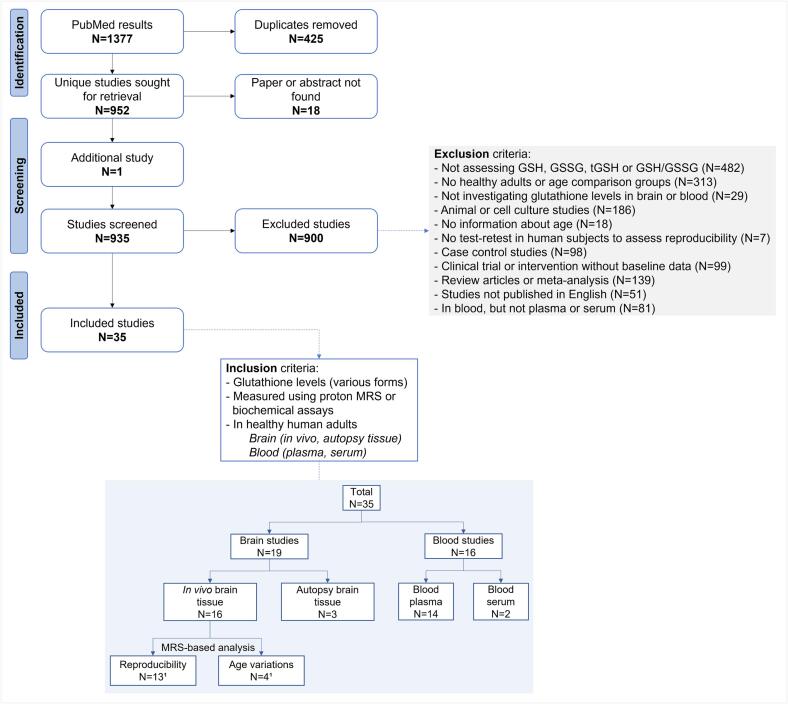
Flowchart for study selection. Note: ^1^one paper is common to both the GSH reproducibility and the GSH variations with age sections, resulting in 16 papers in the *in vivo* MRS category. One additional study was included, independently of our PubMed search (Lim and Xin, 2022). Abbreviation: GSH, glutathione; GSSG, glutathione disulfide; MRS: magnetic resonance spectroscopy; tGSH, total glutathione.

Our flowchart for study selection is provided in Fig. 2.

**Fig. 3:**
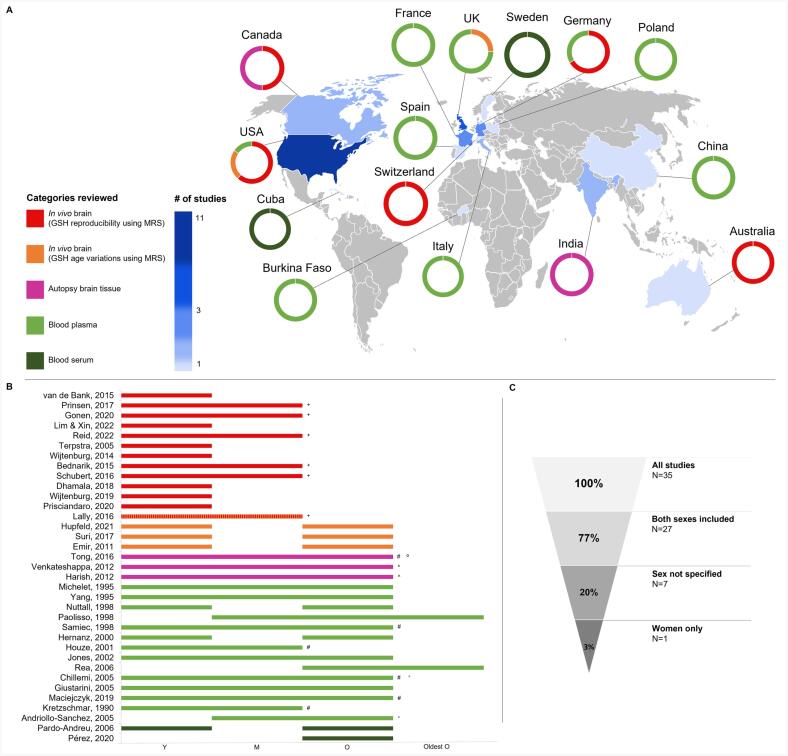
Demographics of studies that met inclusion criteria. A) Geographical distribution of the included studies, per category; B) Age groups, #young and middle-aged groups overlap, such as Kretzschmar et al. (1991), °middle-aged and old groups overlap, such as Chillemi et al. (2005), +not separated into age groups, but instead young to middle-aged as a continuum, such as Prinsen et al. (2017), ^not separated into age groups, but instead young to old as a continuum, such as Harish et al. (2012); and C) Sex distribution of participants grouped by number of studies. Abbreviation: GSH, glutathione; GSSG, glutathione disulfide; MRS: magnetic resonance spectroscopy; tGSH, total glutathione; UK, United Kingdom; USA, United States of America.

**Supplementary figure 1:**
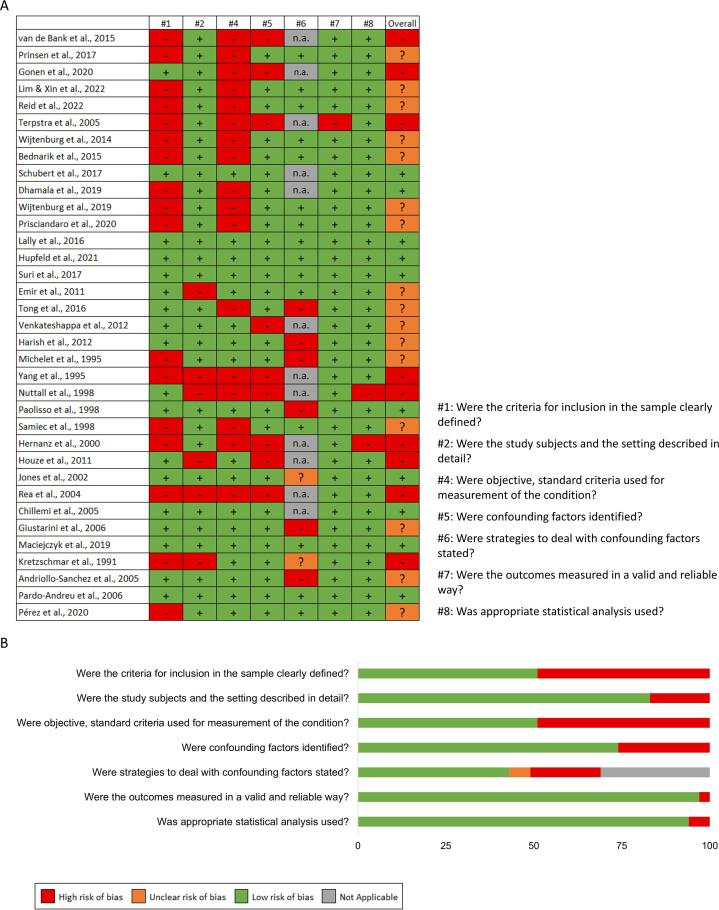


The seven search expressions resulted in 1,377 studies, of which 952 were unique. Of these, 35 studies, totaling 2,100 participants, and publication dates ranging from 1990 to 2022 were included in this review. Fig. 3A maps the geographic distribution of the included studies based on last/senior author affiliation, with most of the publications stemming from North America and Europe. Age groups most contrasted were young versus old adults (as defined in our methods section, N = 10) (Fig. 3B). We also observed that several studies combined age groups, for example, young to middle-aged versus old adults (Fig. 3B). The majority (N = 27, 77%) of studies included both sexes, 20% provided no information on sex, and the remaining investigated GSH levels in females only (Fig. 3C). Supplementary Fig. S1 displays the assessment of the risk of bias of our included studies. We found four items with a risk of bias lower than 70% (description of study subjects and settings; confounding factors identified; valid and reliable outcome measurements; and statistical analysis). The remaining items had a risk of bias of 50% or greater (inclusion criteria; objective measure of the condition; and strategies to deal with confounding factors). The higher risk of bias could be due to the nature of the MRS reproducibility studies (N = 13), and the inclusion of older studies where recruitment of healthy subjects was not as standardized compared to more recent publications.

### 3.2 Findings in brain

#### 3.2.1 In vivo MRS and reproducibility of GSH measurements

In the brain, GSH concentrations assessed using *in vivo* MRS normally range from 1.5 to 3 mM (Harris et al., 2017). GSH has a complex ^1^H-MRS spectrum arising from its three peptide components: glutamate, cysteine and glycine, that contribute to multiple peaks between 2.15 and 4.56 parts per million (ppm) (Harris et al., 2017). Resonances from the glutamate moiety are located at 2.15, 2.55 and 3.77 ppm, from the cysteine part at 2.93, 2.98 and 4.56 ppm, and the glycine moiety contributing to the 3.77 ppm peak (Harris et al., 2017). Each of these peaks forms a multiplet structure (splitting) due to J-coupling (or scalar coupling) between hydrogen nuclei within the whole molecule, thereby reducing the amplitude of the individual peaks and spreading out the signal (Harris et al., 2017). This effect exacerbates overlap of J-coupled resonances with stronger signals, making it difficult to quantify the individual peaks of GSH with conventional MRS approaches, especially at lower field strengths (Harris et al., 2017). A number of spectral editing techniques have been developed to simplify spectra and make quantification of J-coupled resonances more reliable (Harris et al., 2017). Of these, J-difference editing approaches such as MEGA (MEscher-GArwood) (Mescher et al., 1996) combined with a localization scheme such as PRESS (Point-RESolved Spectroscopy) (Bottomley, 1987), together called MEGA-PRESS, have most commonly been used to measure brain GSH (Dwivedi et al., 2020). At higher field strengths (≥3 tesla, T), non-edited approaches have also been applied, relying on peak fitting with prior knowledge of individual metabolite spectra to extract the signal attributable to GSH, using quantification tools such as LCModel (Provencher, 2001). These non-edited approaches have been shown to provide reasonable estimates of GSH (Terpstra et al., 2005, Wijtenburg et al., 2019), with improved performance at very short TEs, due to having less time for both J-coupling related phase dispersion and T2 relaxation (Wijtenburg et al., 2019), thereby preserving signal-to-noise ratio (SNR). At ultra high field strengths (UHF) (≥7T), the impact of J-coupling (which is field independent) becomes smaller compared to the spread of chemical shifts (which increases linearly with field strength), simplifying spectra and making J-coupled resonances easier to quantify. Measuring brain GSH levels using MRS is thus not trivial, and ensuring robust reproducibility of this measure is a prerequisite for the study of age-specific changes.

We identified 13 MRS studies reporting on the reproducibility of GSH measurement in young or young to middle-aged healthy adults (Bednařík et al., 2015, Dhamala et al., 2019, Gonen et al., 2020, Lally et al., 2016, Lim and Xin, 2022, Prinsen et al., 2017, Prisciandaro et al., 2020, Reid et al., 2022, Schubert et al., 2017, Terpstra et al., 2005, van de Bank et al., 2015, Wijtenburg et al., 2014, Wijtenburg et al., 2019) (Table 1), using different magnetic field strengths (i.e., 3T, 4T or 7T). Specifically, six of these studies were conducted at 3T with ROIs in the cingulate (anterior, dorsal anterior, posterior) (Prisciandaro et al., 2020, Wijtenburg et al., 2014), hippocampus (Bednařík et al., 2015), amygdala (Schubert et al., 2017), primary motor (Dhamala et al., 2019), dorsolateral prefrontal (Dhamala et al., 2019), and the medial frontal (including anterior cingulate) (Wijtenburg et al., 2019) cortices, and one was conducted at 4T in the anterior cingulate cortex (Terpstra et al., 2005). The remaining six studies were conducted at 7T in the following ROIs: corona radiata white matter (WM) (van de Bank et al., 2015), and the cingulate (medial pregenual anterior, posterior) (Gonen et al., 2020, Lally et al., 2016, van de Bank et al., 2015), occipital (Prinsen et al., 2017), precuneal (Gonen et al., 2020), motor (Lim and Xin, 2022), medial prefrontal (Lim and Xin, 2022) and/or the dorsolateral prefrontal (Reid et al., 2022) cortices. Compared to lower field strengths, MRS at UHF yields (a) higher SNR, (b) improved spectral resolution (peak separation) due to larger chemical shifts, (c) reduced J-coupling effects (Henning, 2018), and (d) potentially shorter scanning time (Trattnig et al., 2018). In general, these qualities allow for greater sensitivity and more reliable quantification of all metabolites, especially low-concentration metabolites such as GSH (Pradhan et al., 2015). However, these benefits come at the expense of higher susceptibility-induced magnetic field (B_0_) inhomogeneity and radiofrequency excitation (B_1_) inhomogeneity, and elevated radio frequency power deposition. Therefore, studies comparing results across different field strengths are helpful for evaluating benefits of UHF strengths, given the additional technical challenges.

**Table 1:** Reproducibility of GSH measurement using MRS.

**Study**	**Region of interest**	**Field Strength**	**Pulse sequence (TE)**	**Reference**	**Reproducibility measures**	
van de Bank et al., 2015	Corona radiata	7T	Semi-LASER (30 ms)	Water	CV	11.50%
					ICC	53%
	Posterior cingulate				CV	14.40%
					ICC	51%
Lally et al., 2016	Anterior cingulate (medial pregenual)	7T	J-edited PRESS (TE_1_: 37 ms; TE_2_: 69 ms)	Ratio to creatine	CV	Within day 1: 14.95%; Within day 2: 8.54%; Between-session (scan 1): 11.40%; Between-session (scan 2): 8.25%
					ICC	Within day 1: 49%; Within day 2: 88%; Between-session (scan 1): 65%; Between-session (scan 2): 76%
Prinsen et al., 2017	Occipital	7T	STEAM (10 ms)	Water	CV	11.60%
			JDE semi-LASER (72 ms)		CV	7.80%
Gonen et al., 2020	Posterior cingulate Precuneus	7T	STEAM (6 ms)	Water	CV	Within day 1 (scan 1 vs. scan 2): 7.81%; Within day 2 (scan 1 vs. scan 2): 10.69%; Between-session (scan 1): 6.66%; Between-session (scan 2): 9.84%; Overall (4 scans): 10.82%
					ICC	Within day 1 (scan 1 vs. scan 2): 66%; Within day 2 (scan 1 vs. scan 2): 20%; Between-session (scan 1): 72%; Between-session (scan 2): 69%; Overall (4 scans): 68%
				Ratio to creatine	CV	Within day 1 (scan 1 vs. scan 2): 8.99%; Within day 2 (scan 1 vs. scan 2): 6.67%; Between-session (scan 1): 5.69%; Between-session (scan 2): 10.13%; Overall (4 scans): 8.38%
					ICC	Within day 1 (scan 1 vs. scan 2): 33%; Within day 2 (scan 1 vs. scan 2): 59%; Between-session (scan 1): 67%; Between-session (scan 2): 13%; Overall (4 scans): 59%
Lim & Xin, 2022	Primary motor (M1) Prefrontal medial	7T	MEGA- sSPECIAL (80 ms)	Absolute quantification	CV	Motor cortex: 8.60%; Medial prefrontal cortex: 12.80%
			sSPECIAL (16 ms)			Motor cortex: 8.80%; Medial prefrontal cortex: 10.20%
Reid et al., 2022	Prefrontal dorsolateral	7T	STEAM (5 ms)	Water	CV	Intrasubject: 11.90%; Intersubject: 6.50%
Terpstra et al., 2005	Anterior cingulate	4T	STEAM (5 ms)	n.p.	*r*	0.50
Wijtenburg et al., 2014	Anterior cingulate Posterior cingulate	3T	PR-STEAM (6.50 ms)	Water	CV	5.70%
					ICC	42%
					CV	8.60%
					ICC	51%
Bednařík et al., 2015	Hippocampus	3T	Semi-LASER (28 ms)	Water	CV	<20%
Schubert et al., 2017	Amygdala	3T	SPECIAL (6 ms)	Water	n.p.	n.p.
Dhamala et al., 2019	Prefrontal dorsolateral	3T	MEGA-PRESS (68 ms)	Absolute quantification	*r*	MEGA-PRESS (difference edited spectrum) vs. MEGA-PRESS (processed edit-off spectrum): 0.64
	Primary motor (M1)		SPECIAL (8.50 ms)			SPECIAL vs. MEGA-PRESS (processed edit-off spectrum): 0.81; SPECIAL vs MEGA-PRESS (difference edited spectrum): 0.61
Wijtenburg et al., 2019	Medial frontal	3T	PRESS (30 ms)	Water	CV	5.80%
			MEGA-PRESS (120 ms)			13.50%
			PR-STEAM (6.50 ms)			5.40%
			SPECIAL (8 ms)			8%
Prisciandaro et al., 2020	Anterior cingulate (dorsal)	3T	HERMES (80 ms)	n.p.	CV	19.04%
			MEGA-PRESS (120 ms)			7.25%

A total of 13 unique ROIs were assessed in reproducibility studies, including three different regions of the anterior cingulate cortex (medial pregenual, dorsal, and total). CV: ≤10%, excellent; 10–20%, good; 20–30%, acceptable; >30%, poor. ICC: 0.75–1, excellent; 0.60–0.74, good; 0.40–0.59, fair. Abbreviation: CV, coefficient of variation; ICC, intraclass correlation coefficient; JDE semi-LASER, J-Difference Editing semi-Localized by Adiabatic SElective Refocusing sequence; ms, milliseconds; n.p., not provided; PRESS, Point RESolved Spectroscopy; PR-STEAM, Phase Rotation STimulated Echo Acquisition Mode; *r*, Pearson’s correlation coefficient; semi-LASER, semi-Localized by Adiabatic SElective Refocusing sequence; SPECIAL, SPin Echo full Intensity Acquired Localized; STEAM, STimulated Echo Acquisition Mode; T, tesla; TE, echo time.

Reproducibility statistics used were as follows: coefficient of variation (CV, 77%, 10/13), the intraclass correlation coefficient (ICC, 31%, 4/13) and Pearson’s correlation coefficient (*r*, 15%, 2/13) (Table 1; Supplementary Text S1). Reproducibility ranged from good to excellent, with (a) three studies at 3T (Bednařík et al., 2015, Dhamala et al., 2019, Schubert et al., 2017), one study at 4T (Terpstra et al., 2005) and one study at 7T (van de Bank et al., 2015) reporting good reproducibility, (b) one study at 7T (Reid et al., 2022) reporting excellent reproducibility, and (c) three studies at 3T (Prisciandaro et al., 2020, Wijtenburg et al., 2014, Wijtenburg et al., 2019) and four studies at 7T (Gonen et al., 2020, Lally et al., 2016, Lim and Xin, 2022, Prinsen et al., 2017) reporting good to excellent reproducibility. However, it should be noted that of the 13 studies, only seven focused solely on GSH reproducibility along with other low signal metabolites, specifically the 4T study (Terpstra et al., 2005), half (3/6) of the 3T studies (Prisciandaro et al., 2020, Wijtenburg et al., 2014, Wijtenburg et al., 2019), and three 7T studies (Gonen et al., 2020, Lally et al., 2016, Prinsen et al., 2017). Reproducibility values were lower for (a) GSH compared to metabolites easier to detect, such as N-acetylaspartate, myo-inositol, and total choline and creatine (Bednařík et al., 2015, Lally et al., 2016, Reid et al., 2022, Schubert et al., 2017, van de Bank et al., 2015), (b) MEGA-PRESS relative to non-edited sequences (e.g., SPECIAL, PRESS, PR-STEAM) with short TEs (Dhamala et al., 2019, Wijtenburg et al., 2019), and (c) HERMES (TE = 80 ms) relative to MEGA-PRESS using a TE optimized for GSH measurement (TE = 120 ms) (Prisciandaro et al., 2020). While non-edited approaches may provide better precision than edited approaches, accuracy and sensitivity-to-change of non-edited approaches for GSH estimation likely depends on having very short TEs and good spectral quality (Wijtenburg et al., 2019). The measures of spectral quality were assessed using SNR (N = 7), linewidth (N = 10) and the Cramér-Rao Lower Bound for GSH, an estimate of the uncertainty of the concentration estimate (CRLB; N = 11) (Supplementary Table S1) (Kreis, 2016).

#### 3.2.2 Age-specific changes in brain GSH levels measured using *in vivo* MRS

Using MRS, four studies investigated age-specific alterations in GSH levels in the brain of healthy, cognitively unimpaired adults (Emir et al., 2011, Hupfeld et al., 2021, Lally et al., 2016, Suri et al., 2017). Two of these studies were conducted at 3T, using SPECIAL (Suri et al., 2017) and HERMES (Hupfeld et al., 2021), while the other two were at higher magnetic field strengths of 4T (Emir et al., 2011) and 7T (Lally et al., 2016), using MEGA-PRESS (Emir et al., 2011) and J-edited PRESS (Lally et al., 2016), respectively (Table 2). Using 3T, Hupfeld et al. (2021) found higher GSH levels in the medial frontal and the sensorimotor cortices in old compared to young adults. The authors suggested that the observed age-specific increases in regional brain GSH levels may point to a compensatory response to elevation in ROS production to prevent a state of OS (Hupfeld et al., 2021). The remaining three studies reported GSH level decreases with age, in particular, (a) decreases in old versus young adults in the precuneus and the posterior cingulate at 3T (Suri et al., 2017), and the occipital cortex at 4T (Emir et al., 2011), and (b) a negative correlation with age in the medial pregenual anterior cingulate cortex in young to middle-aged adults (20–54 yrs) at UHF (7T) (Lally et al., 2016). The latter study also investigated sex-specific differences in GSH levels and reported higher GSH levels in women compared to men (Lally et al., 2016). It should be noted that the studies performed using edited sequences (Emir et al., 2011, Hupfeld et al., 2021, Lally et al., 2016, Suri et al., 2017) used long TEs, and therefore, possible age-related differential changes in T2 relaxation time of GSH relative to the water or creatine quantitation reference could have confounded estimations of age-related concentration differences.

**Table 2:** Age-specific changes in brain glutathione levels in healthy adults.

**Study**	**Modality**	**Region of interest**	**Method**	**Metabolite**	**Direction of change in the metabolite levels**		**N** (**Y**; **M**; **O**)
Hupfeld et al., 2021	3T MRS	Medial frontal	HERMES	GSH	Old vs. young	in old	60 (37 **Y**; 23 **O**)
		Sensorimotor (lower limb)			Old vs. young	in old	
Suri et al., 2017	3T MRS	Precuneus	SPECIAL	GSH	Old vs. young	in old	147 (30 **Y**; 117 **O**)
		Posterior cingulate			Old vs. young	in old	
Emir et al., 2011	4T MRS	Occipital	DEW MEGA-PRESS	GSH	Old vs. young	in old	44 (22 **Y**; 22 **O**)
Lally et al., 2016	7T MRS	Anterior cingulate (medial pregenual)	J-edited PRESS	GSH	Young to middle-aged	in middle-aged	26 (**Y** to **M**)
Tong et al., 2016	Biochemical assay (autopsy brain tissue)	Frontal (BA9: prefrontal dorsolateral)	HPLC	GSH	Old vs. young to middle-aged	in old	40 (**Y** to **O**)
				GSSG		Ø	
		Cerebellum		GSH	Old vs. young to middle-aged	Ø	
				GSSG		Ø	
		Occipital (BA17: primary visual)		GSH	Old vs. young to middle-aged	Ø	
				GSSG		Ø	
		Caudate		GSH	Old vs. young to middle-aged	Ø	
				GSSG		in old	
Venkateshappa et al., 2012	Biochemical assay (autopsy brain tissue)	Hippocampus	Colorimetry	tGSH	Young to old	with age	25 (**Y** to **O**)
		Frontal		tGSH	Young to old	Ø	31 (**Y** to **O**)
		Cerebellum		tGSH	Young to old	Ø	27 (**Y** to **O**)
Harish et al., 2012	Biochemical assay (autopsy brain tissue)	Frontal	Colorimetry	tGSH	Young to old	with age	32 (**Y** to **O**)

Papers ordered based on the magnet strength used. A total of 12 unique ROIs were assessed, including three different regions of the frontal cortex (medial, dorsolateral prefrontal, and total) and two regions of the occipital cortex (primary visual and total). ROIs were colored according to images provided by the authors (4/7). When the ROI image was not provided, we used the consensus anatomical location. The pink color reflects an increase in the GSSG levels, associated with an increase in OS value. Abbreviation: DEW MEGA-PRESS, Double-Editing with MEshcher-GArwood-Point RESolved Spectroscopy; GSH, glutathione; GSSG, glutathione disulfide; HERMES, Hadamard Encoding and Reconstruction of MEGAEdited Spectroscopy; HPLC, high-performance liquid chromatography; **M**, middle-aged; **O**, old; PRESS, Point RESolved Spectroscopy; ROI, region of interest; SPECIAL, SPin Echo full Intensity Acquired Localized; T, tesla; tGSH, total glutathione; **Y**, young.

The relationship between GSH levels and cognitive function, using the Montreal Cognitive Assessment (MoCA), was interrogated by half (2/4) the MRS studies, and no significant association was found in the ROIs investigated, namely medial frontal (Hupfeld et al., 2021), sensorimotor (Hupfeld et al., 2021) and occipital (Emir et al., 2011). However, it should be noted that while MoCA can robustly detect mild cognitive impairment, detection of subtle cognitive impairments requires analyses of its sub-scores (Charest et al., 2020), which were not conducted by the above-mentioned studies. In addition to cognition, one study assessed the relationship between GSH levels and mobility in the sensorimotor and frontal cortices (Hupfeld et al., 2021). They found that higher GSH levels in the sensorimotor cortex significantly associated with poorer motor performance and greater gait variability in old adults (Hupfeld et al., 2021). As noted above, the authors suggest that increased GSH is an adaptive response to increased OS, and that the degree of elevation is related to associated tissue injury in the sensorimotor cortex (Hupfeld et al., 2021). Over time and with increasing OS, mechanisms that promote GSH synthesis may become overwhelmed, leading to decreased GSH levels as GSH is consumed in oxidation and insufficiently replaced. Longitudinal studies looking at GSH as well as markers of tissue injury and dysfunction (e.g., N-acetylaspartate, local atrophy and tissue microstructure markers from MRI) are needed to elucidate this point.

#### 3.2.3 GSH findings in autopsy brain tissue

In the human autopsy brain, almost all glutathione (>98.80%) is in its reduced form, with greater GSH concentrations in the WM (∼1.18 mM) compared to the grey matter (GM) (∼0.83 mM) (Aoyama, 2021). Three included studies in our review investigated age-specific alterations in GSH levels in autopsy brain tissue from healthy, cognitively unimpaired adults, using HPLC (Tong et al., 2016) or colorimetry (Harish et al., 2012, Venkateshappa et al., 2012). Often considered the standard biochemical assay for GSH, HPLC allows for separation and quantification of compounds (e.g., GSH) from a liquid mixture, while colorimetry relies on reagents that undergo color change in presence of analyte (e.g., GSH) (Cooper et al., 2013, Floreani et al., 1997, Santa, 2013, Corporation, 2014, Hickman, 2016).

Unlike the majority of the *in vivo* MRS studies, the brain autopsy studies lacked clear-cut separation of groups by age (as defined in Methods, Section 2.1), but instead investigated old versus young + middle-aged adults (Tong et al., 2016) or young to old adults (Harish et al., 2012, Venkateshappa et al., 2012). Specifically, Tong et al. (2016) investigated both GSH and GSSG levels using HPLC in autopsy tissue from four different brain regions (Table 2). In older adults relative to young + middle-aged adults, they found higher GSH levels in the frontal cortex and higher GSSG levels in the caudate (Tong et al., 2016). Using colorimetry, the other two studies (Harish et al., 2012, Venkateshappa et al., 2012) investigated tGSH in three brain regions, namely the hippocampus (Venkateshappa et al., 2012), the cerebellum (Venkateshappa et al., 2012), and the frontal cortex (Harish et al., 2012, Venkateshappa et al., 2012). Lower tGSH levels in the hippocampus with increasing age were reported by Venkateshappa et al. (2012) (Table 2). In the frontal cortex, while Harish et al. (2012) found tGSH levels to decrease with age, Venkateshappa et al. (2012) reported no significant change (Table 2). The lack of consensus may stem from differences in brain tissue preparation, namely investigation of GSH level in whole brain tissue homogenate (Venkateshappa et al., 2012) versus its cytosolic fraction only (Harish et al., 2012). Two of the three brain tissue studies also investigated sex-specific changes with age, and found no significant difference in GSH (Tong et al., 2016), GSSG (Tong et al., 2016), or tGSH (Venkateshappa et al., 2012) levels.

#### 3.2.4 Section summary

In summary, irrespective of the different pulse sequences employed for MRS, all studies assessing GSH reproducibility reported good to excellent reproducibility in 13 different brain ROIs. Age-specific changes in brain GSH levels were assessed cross-sectionally using MRS in the living brain and/or biochemical assays in autopsy tissue in 10 unique ROIs, of which six (occipital, precuneal, posterior cingulate, anterior cingulate (medial pregenual), medial frontal and prefrontal dorsolateral regions) had been investigated by reproducibility studies with reliable GSH measures demonstrated. The majority of the *in vivo* MRS studies investigating age-specific changes compared brain GSH levels in old versus young adults, with results indicating region-specific GSH decreases (N = 3; precuneus, posterior cingulate and occipital ROIs) or increases (N = 2, medial frontal and sensorimotor ROIs) in old adults. In autopsy brain tissue, only one study measured GSH and reported an increase in levels with age (i.e., young to old adults) (Tong et al., 2016). While GSH levels in the frontal (Hupfeld et al., 2021, Tong et al., 2016) and occipital (Emir et al., 2011, Tong et al., 2016) brain regions were assessed by both MRS and biochemical analyses, a direct comparison of results is not recommended due to a lack of congruency (a) between age distributions investigated and/or (b) the exact ROIs used. Overall, given the limited number of studies assessing age-related changes in brain GSH levels, we recommend additional investigations (a) for all age groups, but in particular, the understudied middle-aged group, and (b) MRS at UHF (i.e., 7T).

### 3.3 Findings in blood

#### 3.3.1 Age-specific changes in GSH levels in plasma

Under normal conditions, GSH levels in blood plasma are usually of 1 to 6 μM (Hakuna et al., 2015). To date, 14 studies have investigated GSH, GSSG, GSH/GSSG and/or tGSH levels in the plasma of healthy adults (Andriollo-Sanchez et al., 2005, Chillemi et al., 2005, Giustarini et al., 2006, Hernanz et al., 2000, Houze et al., 2001, Jones et al., 2002, Kretzschmar et al., 1991, Maciejczyk et al., 2019, Michelet et al., 1995, Nuttall et al., 1998, Paolisso et al., 1998, Rea et al., 2004, Samiec et al., 1998, Yang et al., 1995), with the majority using HPLC (N = 10, 71%) (Chillemi et al., 2005, Giustarini et al., 2006, Hernanz et al., 2000, Houze et al., 2001, Jones et al., 2002, Michelet et al., 1995, Paolisso et al., 1998, Rea et al., 2004, Samiec et al., 1998, Yang et al., 1995), and the remaining using colorimetry (N = 2, 14%) (Kretzschmar et al., 1991, Maciejczyk et al., 2019), enzyme-rate essay (Nuttall et al., 1998) (N = 1, 7%), and spectrophotometry (N = 1, 7%) (Andriollo-Sanchez et al., 2005) (Table 3). Of the three methods used, HPLC and colorimetry have been defined earlier (see Section 3.2.3). Spectrophotometry assesses the intensity of light after going through a sample, to measure the light absorption or the quantity of chemicals in a solution (Spectrophotometry, 2013, Spectrophotometry, 2020). While colorimetry and spectrophotometry are similar methods, the main difference lies in the wavelengths used by the two, with spectrophotometry using a wider range of wavelengths including those outside the visible range (Difference between colorimetry and spectrophotometry, 2011).

**Table 3:** GSH and GSH-associated protein levels in blood of healthy adults.

**Study**	**Blood**	**Method**	**Metabolite**	**Direction of change in the metabolite levels**		**N** (**Y**; **M**; **O**; **C**)	
Michelet et al., 1995	Plasma	HPLC	GSH	Old vs. young	Ø	201 (108 **Y**; 73 **M**; 20 **O**)	
				Old women vs. young women	Ø		
				Old men vs. young men	in old men		
Yang et al., 1995	Plasma	HPLC	GSH	Old vs. young	in old	n.a.	
			GSSG	Old vs. young	Ø		
Paolisso et al., 1998	Plasma	HPLC	GSH/GSSG	Old vs. middle-aged	in old	80 (30 **M**; 30 **O**; 22 **C**)	
				Centenarian vs. middle-aged	in centenarian		
				Centenarian vs. old	in centenarian		
Samiec et al., 1998	Plasma	HPLC	GSH	Old vs. young to middle-aged	in old	46 (19**Y** to **M**; 27 **O**)	
Hernanz et al., 2000	Plasma	HPLC	GSH	Old vs. young	in old	70 (27 **Y**; 43 **O**)	
Houze et al., 2001	Plasma	HPLC	GSH	Young to middle-aged	Ø	100 (**Y** to **M**)	
Jones et al., 2002	Plasma	HPLC	GSH	Young to old	with age	122 (54 **Y**; 40 **M**; 28 **O**)	
			GSSG		with age		
Rea et al., 2004	Plasma	HPLC	GSH	65-79 vs. 80-89 years old	in 80-89	40 (70-79 yrs)	125 **O**
				80-89 vs. >90 years old	in >90	46 (80-89 yrs)	
				65-79 vs. >90 years old	in >90	39 (90+ yrs)	
Chillemi et al., 2005	Plasma	HPLC	GSH	Post- vs. pre-menopausal women	in post-menopausal women	100 (25 **Y** to **M**; 75 **M** to **O**)	
Giustarini et al., 2006	Plasma	HPLC	GSH	Old vs. young	in old	41 (12 **Y**; 10 **M**; 19 **O**)	
Maciejczyk et al., 2019	Plasma	Colorimetry	GSH	Old vs. young to middle-aged	in old	60 (30 **Y** to **M**; 30 **O**)	
Kretzschmar et al., 1991	Plasma	Colorimetry	tGSH	Young to middle-aged vs. young	in old	18 (5 **Y**; 12 **Y** to **M**)	
			GSSG		Ø		
Nuttall et al., 1998	Plasma	Enzyme-rate essay	tGSH	Young vs. old	in old	124 (66 **Y**; 58 **O**)	
Andriollo-Sanchez et al., 2005	Plasma	Spectro-photometry	tGSH	Old vs. middle-aged to old	Ø	387 (188 **M** to **O**; 199 **O**)	
Pardo-Andreu et al., 2006	Serum	Kinetic assay	GSH	Old vs. young	Ø	50 (20 **Y**; 30 **O**)	
			tGSH	Old vs. young	Ø		
			GSSG	Old vs. young	in old		
Pérez et al., 2020	Serum	Mass spectroscopy	tGSH	Older groups	with age	91 **O**	

Papers ordered first on methods used and then on oldest to newest publication date. The pink color reflects an increase in the GSSG levels, associated with an increase in OS value. Abbreviation: **C**, centenarians; GSH, glutathione; GSSG, glutathione disulfide; HPLC, high performance liquid chromatography; n.a., not applicable; **M**, middle-aged; **O**, old; tGSH, total glutathione; **Y**, young.

The majority of GSH studies (N = 5, 71%) reported significantly lower plasma levels with increasing age in adults (18–85 yrs) (Jones et al., 2002), as well as in old adults relative to (a) young adults by the majority of studies (2/3, 67%) (Giustarini et al., 2006, Yang et al., 1995), and (b) young to middle-aged adults in two studies (Maciejczyk et al., 2019, Samiec et al., 1998). Only two GSH studies in old versus young adults reported an exception, namely Michelet et al. (1995) found no significant differences, and Hernanz et al. (2000) demonstrated increased GSH levels. No significant GSH level difference was found between middle-aged and young adults (Houze et al., 2001). Investigating older adult subgroups, Rea et al. (2004) found significantly lower plasma GSH levels in individuals 80–89 yrs old relative to those 65–79 yrs of age, while people >90 yrs of age (the oldest-old) showed higher GSH levels than those 65–79 or 80–89 yrs old.

When investigating sex-specific changes in plasma GSH levels, four studies reported a lack of significant differences between age-matched men and women (Houze et al., 2001, Jones et al., 2002, Michelet et al., 1995, Yang et al., 1995). Specifically, when assessing men and women separately, Michelet et al. (1995) reported significantly lower GSH levels in old relative to young men, but not women, although only age and not menopause status was taken into account for women. Segregating by menopause status in women, Chillemi et al. (2005) found significantly lower GSH levels in post- (50–90 yrs) relative to pre-menopausal women (30–45 yrs). This observation is in line with (a) decreased plasma GSH levels in women who underwent surgical menopause (Kaur et al., 2017), and (b) decreased serum tGSH levels in post- relative to pre-menopausal women (Ramírez-Expósito et al., 2014).

In addition to GSH, 6 studies also assessed plasma GSSG (N = 3) (Jones et al., 2002, Kretzschmar et al., 1991, Yang et al., 1995), GSH/GSSG (N = 1) (Paolisso et al., 1998) and/or tGSH (N = 3) (Andriollo-Sanchez et al., 2005, Kretzschmar et al., 1991, Nuttall et al., 1998) levels. Two studies investigating GSSG reported no significant difference between young and middle-aged adults (Jones et al., 2002, Kretzschmar et al., 1991). Investigating old adults, there was a lack of consensus. Specifically, while one study did not find a significant difference in GSSG levels in old relative to young adults (Yang et al., 1995), a second study reported increasing levels of GSSG (linear relationship) from 45 yrs of age (Jones et al., 2002). Paolisso et al. (1998) investigated GSH/GSSG ratio in centenarians, and ordered them from high to low, as follows: middle-aged adults (<50 yrs) > centenarians (>99 yrs) > older adults (75–99 yrs) (Table 3). Literature suggests that decreased GSH/GSSG ratios reflect OS (Zitka et al., 2012), due to decreased GSH levels, increased GSSG values, or both. The same study also assessed other indices of OS (i.e., reaction products of malondialdehyde with thiobarbituric acid and lipid hydroperoxides), and found an inverse pattern to the GSH/GSSG ratio, namely, older adults > centenarians > middle-aged adults (Paolisso et al., 1998). It has been hypothesized that centenarians tend to have higher GSH levels due to multiple factors such as different genetic predisposition, life habits (e.g., intake of vitamins A, C, and E), and fewer chronic diseases (e.g., cancer) (Mecocci et al., 2000, Paolisso et al., 1998). tGSH levels were (a) significantly decreased in young relative to old adults (Nuttall et al., 1998), (b) significantly decreased in young adults (27–35 yrs) compared to young to middle-aged (36–57 yrs) (Kretzschmar et al., 1991), while (c) no significant differences were observed between middle-aged to old (55–70 yrs) and old (70–85 yrs) adults (Andriollo-Sanchez et al., 2005). However, it should be noted that two (Andriollo-Sanchez et al., 2005, Kretzschmar et al., 1991) out of the three studies investigating tGSH levels did not have a clear separation of age groups, as defined in our methods section.

#### 3.3.2 Age-specific changes in GSH levels in serum

In serum, two studies investigated GSH, GSSG, and/or tGSH in serum of healthy adults, using two techniques (Pardo-Andreu et al., 2006, Pérez et al., 2020) (Table 3). Specifically, the kinetics assay used by Pardo-Andreu et al. (2006), is an enzyme-based method used to continuously assess the activity of a given enzyme in a solution (What are enzyme kinetic assays? An overview, 2021). Considered sensitive and accurate, the method allows one to visually control the reaction rate (Kramer, 1980). Pérez et al. (2020) used mass spectroscopy/spectrometry, which is an analytical way of determining the mass-to-charge ratio or of measuring the molecular weight of a component such as a protein, in biological solutions (Iwasaki et al., 2009, Wang et al., 2022, What is mass spectrometry, 2010). Pardo-Andreu et al. (2006) reported no significant changes in GSH levels in old relative to young adults, but found increased GSSG in old adults, the latter most likely reflecting an increase in OS (Abdalla et al., 1990). With regard to tGSH levels (a) no significant change was observed in old versus young adults (Pardo-Andreu et al., 2006), but (b) a decrease with age was reported in old adult sub-groups (ages 60–66, 72–78, 81–87 and ≥90 yrs) (Pérez et al., 2020). Only Pérez et al. (2020) investigated sex-specific variations in tGSH levels, and found no significant differences.

#### 3.3.3 Section summary

In summary, of the 16 cross-sectional studies in blood, 11 (69%) assessed GSH, with fewer studies (N = 8, 50%) investigating GSSG, GSH/GSSG and/or tGSH. Relative to young adults, GSH levels in peripheral blood were decreased in old adults in the majority (6/9, 67% including age-overlap studies) of studies, indicating an increase in OS with age (Bajic et al., 2019, Zhu et al., 2006). We also found that the GSSG literature, to date, suggested stable levels until middle-age (Jones et al., 2002, Kretzschmar et al., 1991), but additional studies are needed to reach consensus. Results from the five tGSH studies were harder to interpret given that 40% of these studies did not have a clear separation of age groups. Overall, while more studies assessed glutathione in blood than brain, none looked at the association between glutathione and cognition, which we recommend be explored in future studies.

## 4 Discussion

We conducted a comprehensive review of the literature to examine the consistency of age-specific GSH level changes in the brain and peripheral blood across studies.

### 4.1 Age-specific changes in GSH levels in brain and blood

#### 4.1.1 Brain

Obtaining robust insights into age-related brain GSH level variations *in vivo* is dependent on the reproducibility of MRS measurements. However, studies interrogating the reproducibility of GSH measures is a relatively recent phenomenon, with publication dates of the 13 identified studies ranging from 2014 to 2022. This is not surprising given that MRS has been historically used to study (a) high-concentration and easily detectable metabolites at lower field strengths, such as N-acetylaspartate, choline, creatine and myo-inositol (Cleeland et al., 2019, Huibregtse et al., 2021), and (b) low-concentration non-GSH metabolites such as gamma-aminobutyric acid and glutamate (Runia et al., 2022). Demonstration of good to excellent reproducibility of GSH measures by all 13 studies in 13 ROIs distributed throughout the brain (limbic (N = 4), frontal (N = 4), temporal (N = 2), parietal (N = 1), occipital (N = 1), and the corona radiata (N = 1)) adds weight to age-specific GSH level findings.

The literature investigating age-related brain GSH level variations using MRS or biochemical assays is in its infancy, with only seven cross-sectional studies meeting our inclusion criteria. Together, these studies investigated GSH variations in 10 ROIs distributed in the frontal (N = 2), limbic (N = 2), occipital (N = 2), parietal (N = 1), caudate (N = 1), sensorimotor (N = 1) or cerebellar (N = 1) regions. With increasing age, GSH levels were observed to significantly decrease in 40% of the ROIs (4/10; limbic, parietal, and occipital), increase in 30% (3/10; frontal lobe), and remain stable in 30% (3/10; occipital, cerebellum, and caudate). Of the above-mentioned ROIs, GSH levels in the frontal (Hupfeld et al., 2021, Tong et al., 2016) and occipital (Emir et al., 2011, Tong et al., 2016) regions were assessed by both MRS and biochemical analyses. However, a direct comparison of these results was not possible due to a lack of congruence between the (a) age groups investigated, namely, clear-cut young and old groups (MRS (Emir et al., 2011, Hupfeld et al., 2021)) versus young + middle-aged and old groups (biochemical assay (Tong et al., 2016)), and/or (b) specific ROIs used: medial frontal (Hupfeld et al., 2021) versus prefrontal dorsolateral cortex (Tong et al., 2016), and occipital cortex (Emir et al., 2011) versus primary visual cortex (Tong et al., 2016). These factors may explain why occipital GSH levels were reported to be decreased using MRS (Emir et al., 2011) and stable using HPLC (Tong et al., 2016).

Moreover, our findings suggest that variations with age of GSH levels in the human brain are region-specific. Lending strength to this observation is the large body of literature on the region-specific impact of age on structural and functional brain imaging measures in human (Feng et al., 2020, Pomponio et al., 2020). Our observation is also supported by the recently published metabolome atlas of the aging mouse brain (N = 10 ROIs, four age groups) that demonstrates region-specific variations in glutathione levels when investigating (a) single time-points, as well as (b) age-specific changes (Ding et al., 2021). In addition, spatiotemporal ribonucleic acid-sequencing (RNA-seq) analyses in the mouse brain (N = 15 ROIs, seven age groups) further demonstrate region-specific (a) expression of *Gss*, *Gclc* and *Gclm*, three genes encoding enzymes involved in GSH synthesis, at a single time point, as well as (b) change in the expression level of these genes with age, with examples that include age-related increases of *Gclc* and *Gclm* in the hypothalamus and medulla, respectively (Hahn et al., 2022, Spatiotemporal Brain Map, 000). Given these multiscale findings, spatiotemporal level changes in brain GSH and other antioxidants may indicate that certain brain regions, in particular, metabolically expensive hub regions (e.g., posterior cingulate cortex) (Badhwar et al., 2017, Tomasi et al., 2013), are more vulnerable to aging-related OS (Badhwar et al., 2017, Hahn et al., 2022, Tomasi et al., 2013). Indeed, brain hub regions in humans do exhibit a spatiotemporally distinctive transcriptomic pattern dominated by genes associated with metabolic processes, including those with oxidoreductase activity (e.g., GPx) (Xu et al., 2022).

##### 4.1.1.1 Considerations for age-specific brain GSH level investigations

**MRS analyses:** It should be noted that both WM and GM volume decline with age, with some studies reporting GM volume declining faster with age, particularly in prefrontal areas (MacDonald and Pike 2021). As with most MRS-visible metabolites in the healthy brain, GSH is not detectable in cerebrospinal fluid (CSF) (Near et al., 2021). The use of GSH/total creatine (tCr) ratios thus intrinsically corrects for differing CSF content, with the caveat that tCr concentration may also change with age. Absolute quantitations of GSH referenced to unsuppressed water, however, would be lower for voxels containing CSF, such as a midline posterior cingulate cortex voxel, and so tissue partial volume correction is required, and is typically performed. Therefore, increased CSF content due to age-related atrophy would generally be compensated for. Regarding differences in GSH concentrations in GM vs. WM, a literature search produced 6 relevant papers. Two reported significantly higher GSH in GM vs. WM (Chan et al., 2019, Srinivasan et al., 2010), three reported no difference in GSH or GSH/tCr between GM and WM (Bhogal et al., 2020, Ganji et al., 2014, van de Bank et al., 2015), and one reported a numerically higher GSH concentration in WM but did not indicate significance (An et al., 2015). Thus, while evidence for differences in MRS-visible GSH in GM vs. WM is equivocal, it would be prudent in future studies to account for differences in GM and WM volume fractions with age when using voxels with a mixture of tissues, either via a more involved partial volume correction based on data from multiple voxels, or statistically controlling for either GM or WM fraction.

**Biochemical analyses of autopsy brain tissue:** A series of physical and chemical changes take place in the body following death (reviewed by Brooks, 2016). Relevant to brain tissue, perturbations (increases or decreases) in levels of several metabolites, including those associated with GSH (e.g., glycine), have been reported 2–5 h post mortem (Gonzalez-Riano et al., 2017). tGSH content has also been reported to decrease rapidly after only 10 min, and was only half its original content after four hours in biopsy specimens from superficial brain tissue in human (Perry et al., 1981). This may be due to the rapid metabolization of GSH during the collection, freezing, and fixation interval, as demonstrated using mouse brain (Miller et al., 2009). Therefore, a main technical consideration for brain autopsy GSH measurements is having a short post-mortem interval for collection of brain tissue as this allows for more accurate measurements. Unfortunately, short delays are not always possible for human studies, and our included studies report post-mortem intervals ranging from 2.5 to 27 h (Supplementary Table S2).

#### 4.1.2 Blood

Glutathione levels (both reduced and oxidized forms) in the liquid portion of blood can act as a readout of systemic (whole body) OS (Anderson and Meister, 1980). In our review, the most investigated contrast in blood was old versus young adults (10/16), with a decrease in GSH levels reported in older adults by the majority of studies (6/9; 67%). Two studies in blood investigated the oldest-old (Paolisso et al., 1998, Rea et al., 2004), an age-group composed of individuals 90 yrs of age and older (Paganini-Hill et al., 2016). GSH levels and the GSH/GSSG ratio were reported to be higher in these individuals relative to those less-old (i.e., 60–90 yrs), indicating that people who survive to reach the oldest-old category may have an overall more efficient antioxidant profile (Traverso et al., 2003). The latter promotes a state of lower OS, which in super-agers has been associated with superior memory (Mapstone et al., 2017). While middle-aged individuals were included in 11/16 studies, the majority (6/11) of these studies lacked a clear separation of age-groups (as defined in our methods section), making interpretation of results challenging.

##### 4.1.2.1 Considerations for age-specific blood GSH level investigations

Moreover, in our review, plasma, and not serum, was the liquid blood portion used by the majority (88%) of studies to interrogate age-specific changes in GSH levels. This preference for plasma may be attributed to some of its advantages over serum, such as a larger volume, no clotting time delay and a lower risk of haemolysis or the breakdown of erythrocytes (Uges, 1988). In fact, contamination from degrading/leaking erythrocytes can artificially increase GSH and GSSG levels in both plasma and serum, given that these cells contain approximately two orders of magnitude higher glutathione than the liquid portion of blood (Tomin et al., 2021). A linear increase in both plasma GSH and GSSG levels was demonstrated for blood left at room temperature over a three hour observation period, with significant increases becoming evident as early as one hour post-collection (Tomin et al., 2021). Ten of 16 (63%) blood studies in our review reported the time to processing of plasma or serum, and all performed this step within 20 min of blood draw (Supplementary Table S3), thereby minimizing glutathione contamination from erythrocytes. Oxidation of GSH during sample preparation (Nuhu et al., 2020, Tomin et al., 2021) can also lead to an under-estimation of GSH and an over-estimation of GSSG concentrations (Nuhu et al., 2020). While this error can be counteracted by the use of thiol-blocking (alkylating) agents (Tomin et al., 2021), this information was not clearly stated by the majority of studies included in our review. Given that accurate biochemical measures of glutathione (both reduced and oxidized forms) in blood are crucially dependent on proper blood-collection protocol and pre-analytical processing factors, we recommend that these details be provided by studies.

### 4.2 GSH and clinical trials

**Supplementary figure 2:**
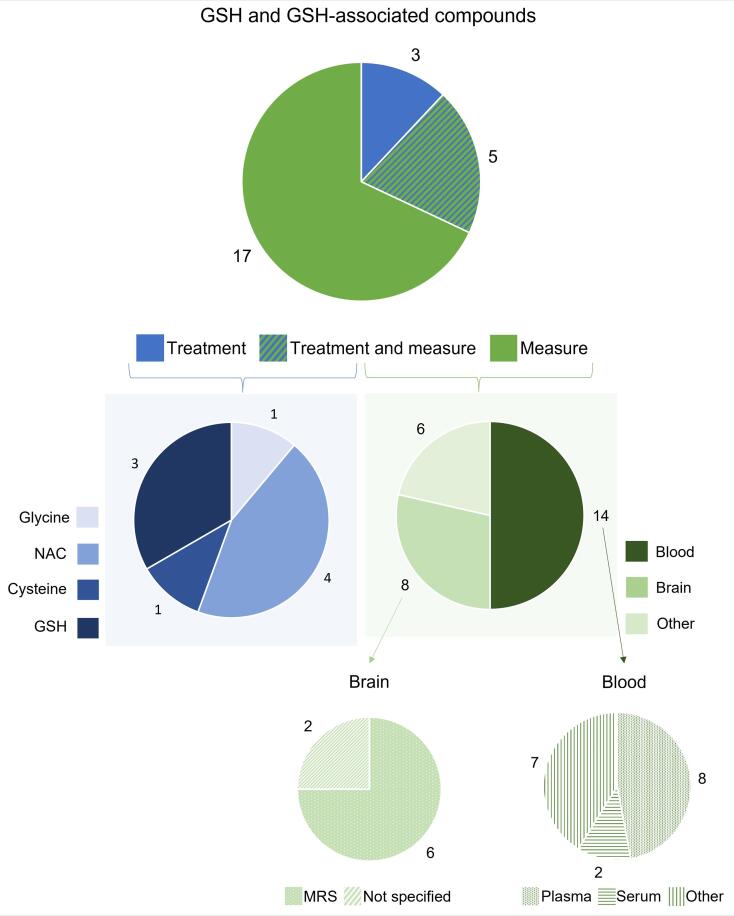


**Supplementary figure 3:**
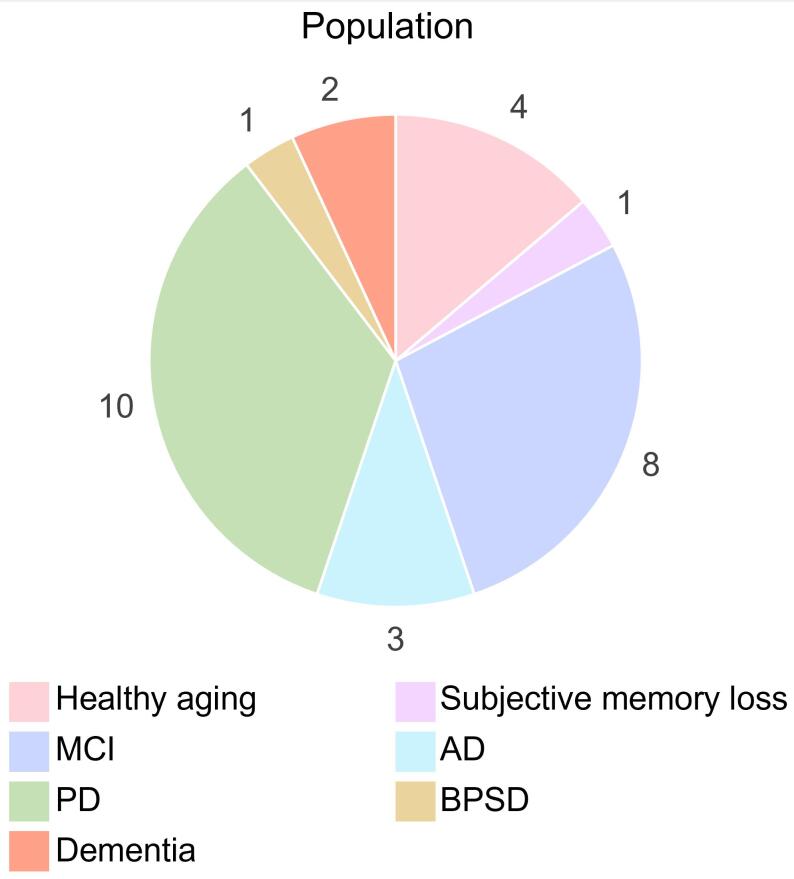


The effects of increased OS levels with age have been found to be reversible in rodents, motivating efforts to find the means to promote similar effects in humans (Ghoneum et al., 2020, Suh et al., 2001). Measures of GSH have been or are currently being used by a variety of intervention studies aimed at promoting antioxidant levels, in order to reduce OS due to aging and neurodegenerative diseases. Our search on *clinicaltrials.gov* (ClinicalTrials.gov) identified 25 trials using GSH and associated compounds (specifically tGSH, GSH/GSSG, GPx and GR) as a treatment (3/25), an outcome measure (17/25), or both (5/25) (Supplementary Fig. S2, Supplementary Table S4). Specifically, treatments comprised dietary supplements (N = 13), drugs (N = 8), physical exercise (N = 5) and aromatherapy (N = 1). Four of these treatment trials involved healthy individuals (Dairy Intake, 0000, Effects of aRecreational, 0000, Effects of Blueberry, 0000, The Safety, 0000) (Supplementary Fig. S3), with two reporting higher GSH levels in brain (Choi et al., 2015, Dairy Intake, 0000) and in blood (The Safety, 0000, Xue et al., 2022) following dietary supplements intake (i.e., RiaGev™, dairy food), thereby indicating that an increase in GSH levels is possible post-intervention in human.

## 5 Conclusion

GSH is one of the most abundant antioxidants that helps fight and prevent damage caused by OS (Kwon et al., 2019), and is thought to play an important role in both aging and age-related neurodegenerative diseases (Dix et al., 2017). Our review highlighted that the investigation of GSH levels in healthy aging is emergent, as evidenced by the limited number of studies in each category: brain (N = 13 reproducibility, N = 7 age-specific variations), blood (N = 16). Overall, the literature suggests that aging is associated with increased OS in brain and body, but the timing and regional distribution of changes requires further study. In addition, since OS in midlife and beyond may contribute to accelerated aging, it would be informative for future studies to incorporate measures of “brain age” derived from MRI in addition to chronological age (Elliott et al., 2019). Given that GSH levels in the brain may also vary due to polymorphisms in GSH-associated genes (e.g., different *Gclc* polymorphisms (Xin et al., 2016)), we recommend that future studies also take genotype into consideration. Our review also identified that longitudinal studies, and studies assessing GSH levels in both brain and blood are lacking. Moreover, while several (46%) reproducibility studies in our review were conducted at 7T, a field strength better suited for the detection of GSH (Balchandani and Naidich, 2015, Godlewska et al., 2017), only one study assessed age-specific GSH level variations at this magnet strength. As access to 7T scanners improves with time, we expect that more GSH studies will be conducted at UHF in future, improving the number and quality of *in vivo* studies of GSH in aging. This will allow us to better understand the role of OS towards aging well or poorly, provide a reference to assess whether OS is accelerated in neurodegenerative diseases of aging, and provide objective measures to assess the efficacy of interventions to reduce OS.

## Funding

This work was supported by the Fonds de Recherche Québec - Santé (FRQS) [Bourse de formation de maîtrise, 2022–2023] (F.D.); FRQS [Chercheur boursiers Junior 1, 2020–2024], Fonds de soutien à la recherche pour les neurosciences du vieillissement from the Fondation Courtois and the Quebec Bio-Imaging Network [#PP 19.20] (A.B.); and the Canadian Institutes of Health Research grant [#153005] (S.N. (grantee), S.S.).

## Declaration of Competing Interest

The authors declare that they have no known competing financial interests or personal relationships that could have appeared to influence the work reported in this paper.

## Data Availability

No data was used for the research described in the article.
