# Supplementary material for: Changes in levels of the antioxidant glutathione in brain and blood across the age span of healthy adults: A systematic review
Source: Neuroimage Clin. 2023 Aug 26;40:103503. doi: 10.1016/j.nicl.2023.103503 (PMC10520675; doi:10.1016/j.nicl.2023.103503)
Supplement: Supplementary data 1 [file mmc1.docx]

**Supplementary Material**

**S1. Reproducibility statistics**

**Coefficient of variation and intraclass correlation coefficient**

Two measures of variability are often used to assess reproducibility of repeated measurements, the coefficient of variation (CV), and the intraclass correlation coefficient (ICC) [(Walters et al., 2020)](https://paperpile.com/c/Cb0l4z/oVo8t).

CV: The CV (sometimes abbreviated as CoV) provides a relative measure of the dispersion of the data and is calculated as the ratio of the standard deviation of a measure to the mean (usually in a population) [(Trinidad, 2020)](https://paperpile.com/c/Cb0l4z/A6ukw). When applied to reproducibility assessments (paired replicate measurements), however, the CV is calculated differently, most commonly using the root-mean-square method [(Hyslop and White, 2009)](https://paperpile.com/c/Cb0l4z/sTO1a):

$$\mathrm{CV}\left( \% \right)=100\times\sqrt{\frac{\Sigma{\frac{(d}{m)}}^{2}}{2n}}$$

where d is the difference and m is the mean of each paired measurement, calculated over n pairs. Other formulations, such as the logarithmic method [(How should I calculate a within-subject coefficient of variation?, 2006; Martin Bland and Altman, 1996)](https://paperpile.com/c/Cb0l4z/Z8ZWy+fhJue) and within-subject standard deviation method [(Jones and Payne, 1997; Synek, 2008)](https://paperpile.com/c/Cb0l4z/tfmSx+965eb) can also be used. In the context of metabolite concentration estimates of low-concentration metabolites, such as GSH, from MRS, a CV ≤10% was considered to represent excellent reproducibility, a CV between 10-20% was considered good, between 20-30% was considered acceptable, and >30% considered poor.

ICC: The ICC provides a measure of the similarity between measurements of the same quantity. While there are multiple forms of the ICC for different applications, the one most appropriate for assessing consistency of MRS concentration estimates from multiple scans of the same set of individuals using the same sequence/scanner (test/retest) would use a two-way mixed effects model, absolute agreement as the type of relationship, and mean of ratings as the unit [(Koo and Li, 2016)](https://paperpile.com/c/Cb0l4z/pvfmM). Reproducibility is considered excellent when the ICC is between 1 and 0.75, good when 0.75 > ICC ≥ 0.60, fair when 0.60 > ICC ≥ 0.40 and poor when 0.40 > ICC [(Cicchetti, 1994)](https://paperpile.com/c/Cb0l4z/yn4D4).

**Pearson’s correlation coefficient**

Commonly used in statistics is Pearson’s correlation coefficient or r, to assess the strength of a relationship between two variables [(Correlation coefficient: Simple definition, formula, easy steps, 2021)](https://paperpile.com/c/Cb0l4z/2qOy8). A value of r close to 1 indicates a strong positive relationship, a value of -1 reflects a strong negative relationship, and a value of 0 indicates no correlation between the variables [(Correlation coefficient: Simple definition, formula, easy steps, 2021)](https://paperpile.com/c/Cb0l4z/2qOy8).

**S2. Supplementary figures**

**
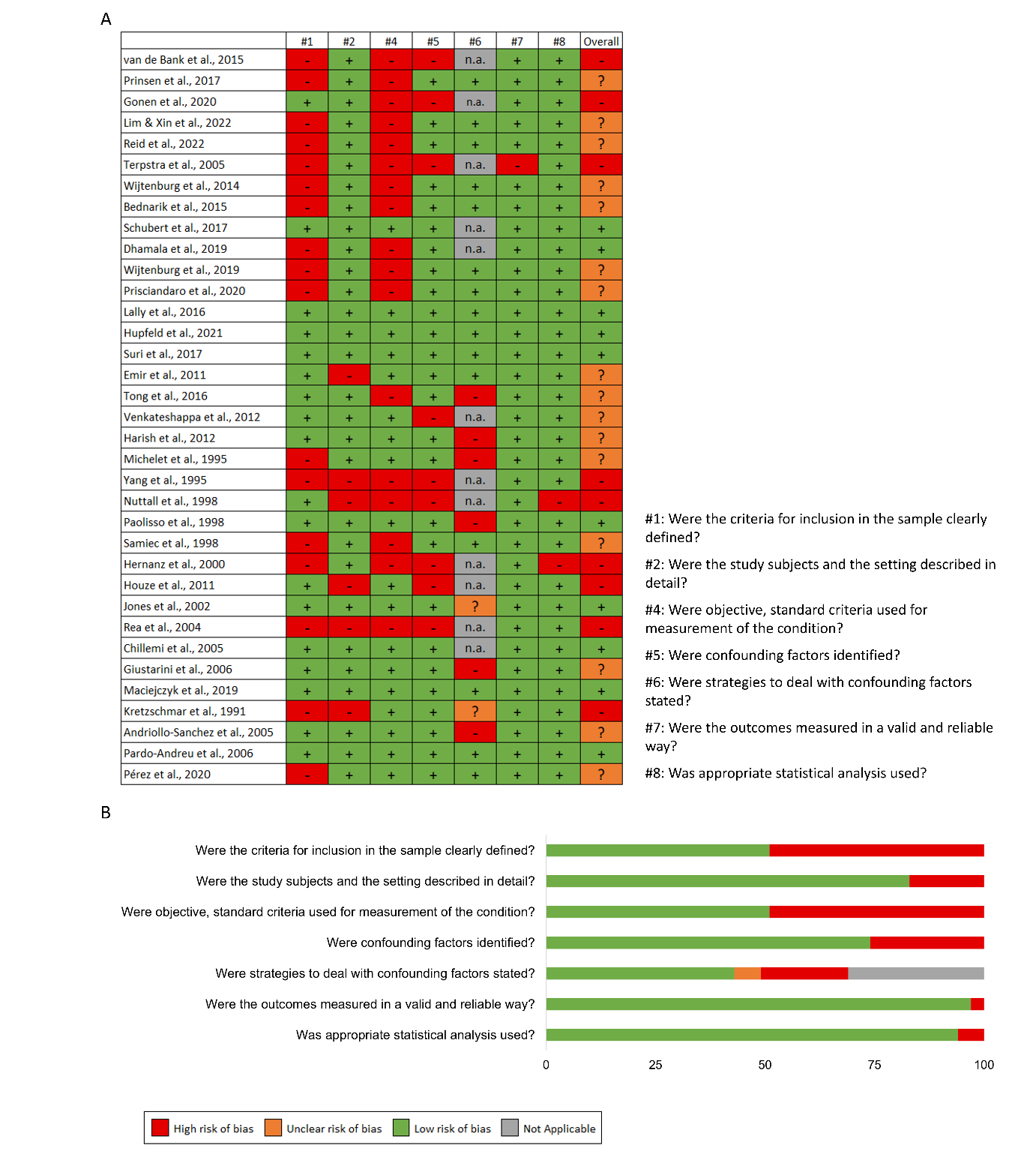
Supplementary Figure S1: Assessment of risk of bias**

A) Risk of bias for each included study; and B) Risk of bias graph displaying the author’s judgment about each question for the risk of bias as percentages, per item. Question #3 from JBI’s checklist was dismissed after being marked as “Not Applicable” for all studies, due to the nature of the question not related to our work. Color code used in the “Overall” column: green = all green across the row except one; orange = all green across the row except two; red = all green across the row except for three or more boxes. If column “#5” (identification of confounding factors) was “No”, column “#6” (dealing with identified confounding factors) was “n.a.”. Abbreviation: JBI, Joanna Briggs Institute; n.a., not applicable.


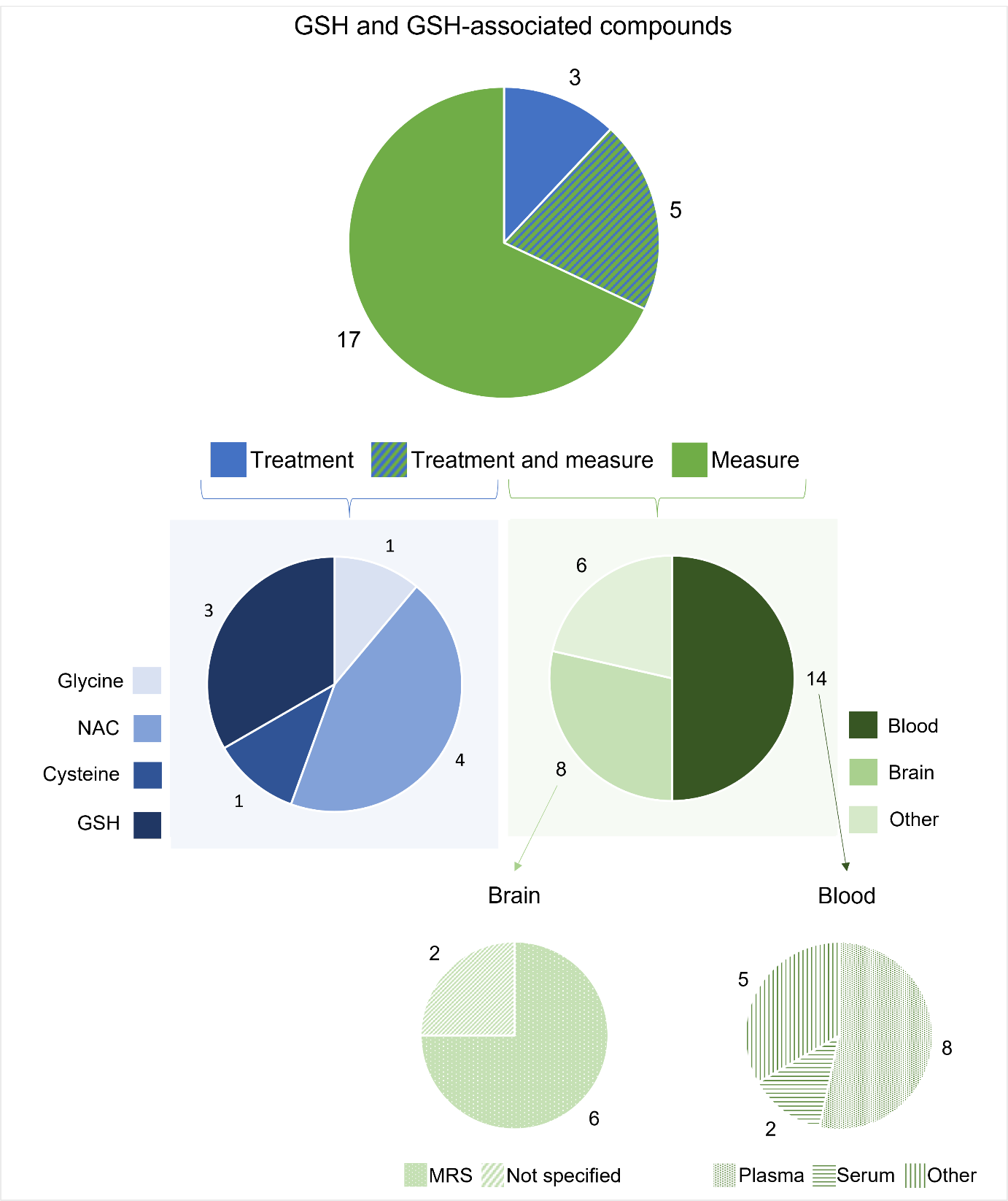


**Supplementary Figure S2: Information about included clinical trials**

The keyword “Glutathione” was used in association with (a) “Healthy Aging”, (b) “Mild Cognitive Impairment”, (c) “Amnestic Mild Cognitive Impairment”, (d) “Alzheimer’s Disease”, (e) “Parkinson’s Disease”, (f) “Lewy Body Dementia”, (g) “Frontotemporal Dementia”, and (h) “Vascular Dementia”, on the website clinicaltrials.gov. 25 were relevant to our review. Searches conducted up to December 2022. Other measures included muscle tissue biopsy, urine, cerebrospinal fluid, and not specified. In blood, “other” represents clinical trials where the part of blood was not specified. Abbreviation: GSH, glutathione; MRS, magnetic resonance spectroscopy; NAC, N-acetylcysteine.


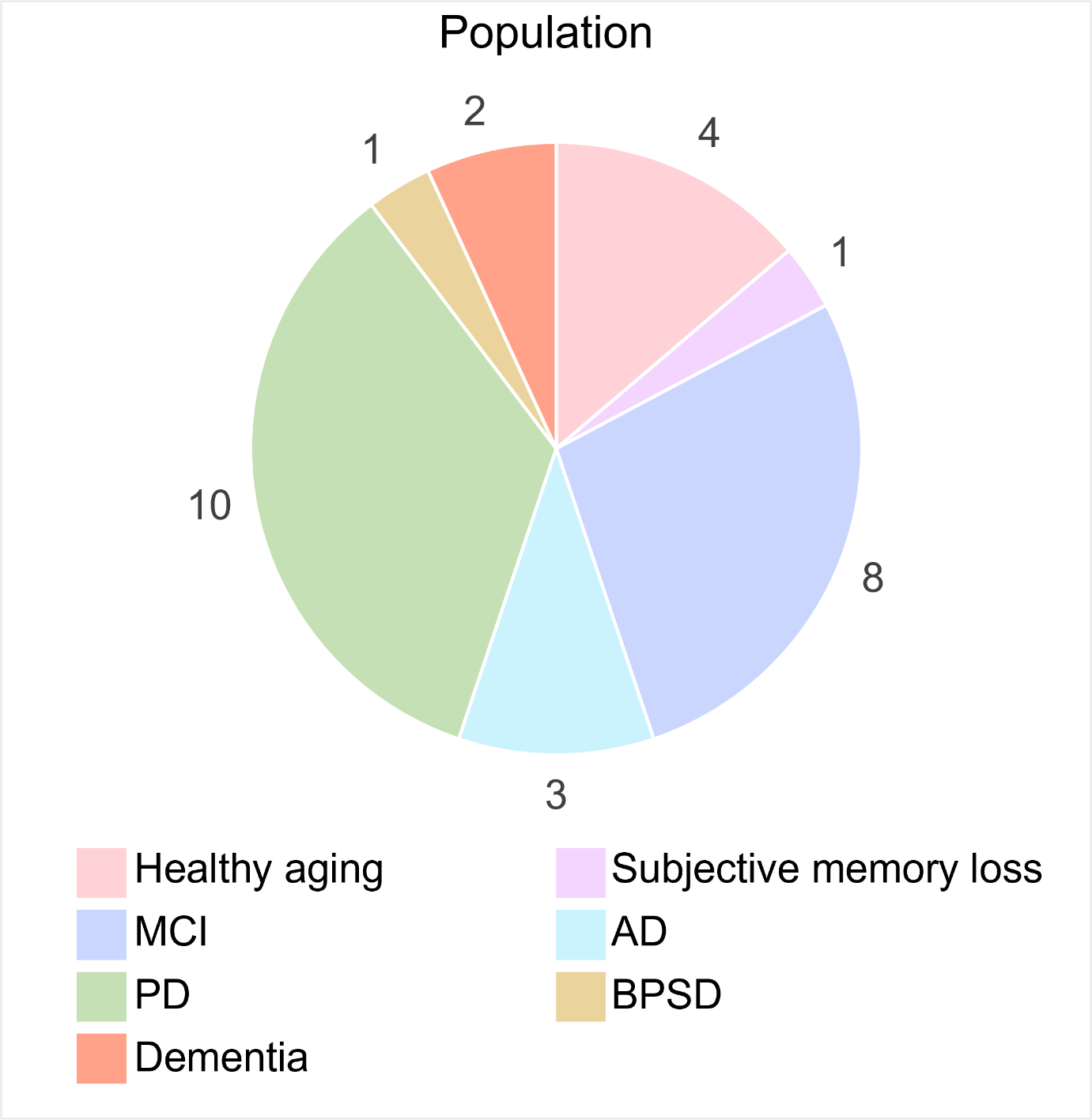


**Supplementary Figure S3: Different participant groups included in clinical trials**

Abbreviation: AD, Alzheimer’s disease; BPSD, behavioral and psychological symptoms of dementia; MCI, mild cognitive impairment; PD, Parkinson’s disease.

**S3. Supplementary tables**

| **Study** | **GSH CRLB (%)** | **SNR**  **(Method)** | **Linewidth (Hz)** |
| --- | --- | --- | --- |
| van de Bank et al., 2015 | Corona radiata: 12.60  Posterior cingulate: 13.20 | n.p. | n.p. |
| Lally et al., 2016 | Day 1/Scan 1: 6.14 ± 2.20  Day 1/Scan 2: 6.13 ± 1.38  Day 2/Scan 1: 5.83 ± 2.25  Day 2/Scan 2: 5.35 ± 0.82 | n.p. | 12.14 ± 1.48 |
| Prinsen et al., 2017 | STEAM: 5.70  JDE semi-LASER: 3 | n.p. | STEAM: 10  JDE semi-LASER:  12.2 ± 1.2 |
| Gonen et al., 2020 | 5.0 ± 0.8 | n.p. | n.p. |
| Lim & Xin, 2022 | MEGA-sPECIAL: 19.50  sSPECIAL: 7 | sSPECIAL: motor cortex: Scan 1: 548 ± 51, Scan 2: 587 ± 86; Medial prefrontal: Scan 1: 486 ± 59, Scan 2: 504 ± 32  MEGA-sSPECIAL-edit-off: motor cortex: Scan 1: 235 ± 60, Scan 2: 236 ± 53; Medial prefrontal: Scan 1: 257 ± 34, Scan 2: 272 ± 28  (Height of the NAA peak at 2.02 ppm divided by the standard deviation of the noise between 9.5 and 10 ppm) | sSPECIAL: motor cortex: Scan 1: 12 ± 0.80, Scan 2: 11.90 ± 0.40; Medial prefrontal: Scan 1: 12.60 ± 1.10, Scan 2: 13.70 ± 2.10  MEGA-sSPECIAL: motor cortex: Scan 1: 12.40 ± 0.90, Scan 2: 12.10 ± 1; Medial prefrontal: Scan 1: 12.70 ± 0.70, Scan 2: 12.50 ± 1.20 |
| Reid et al., 2022 | Scan 1: 4.30  Scan 2: 4.70 | Scan 1: 58.80 ± 7.50  Scan 2: 58.30 ± 12.60  (LCModel S/N) | Scan 1: 15.60 ± 2.60  Scan 2: 15.30 ± 1.90 |
| Terpstra et al., 2005 | 14 | High (value not provided) | 6-11 (average: 8) |
| Wijtenburg et al., 2014 | Anterior cingulate: Scan 1: 6.40; Scan 2: 6.40  Posterior cingulate: Scan 1: 7.60; Scan 2: 8.20 | Anterior cingulate: Scan 1: 36.60; Scan 2: 33.50  Posterior cingulate: Scan 1: 32.30; Scan 2: 31.10  (LCModel S/N) | Anterior cingulate: Scan 1: 0.02; Scan 2: 0.03  Posterior cingulate: Scan 1: 0.02; Scan 2: 0.02 |
| Bednařík et al., 2015 | <20 | 53.60 ± 8.10  (Amplitude of the NAA resonance at 2.02 ppm, divided by the root-mean-square of the noise on the summed spectrum) | 9 |
| Schubert et al., 2017 | 24 ± 11 | 21 ± 5  (LCModel S/N) | 8 |
| Dhamala et al., 2019 | n.p. | MEGA-PRESS: prefrontal dorsolateral: 337 ± 57; primary motor: 290 ± 56  SPECIAL: prefrontal dorsolateral: 693 ± 80; primary motor: 607 ± 87  (Amplitude of the NAA peak, divided by the standard deviation of the noise within spectral windows [−1.4, −0.4] ppm and [9, 10] ppm) | MEGA-PRESS: prefrontal dorsolateral: 4.40 ± 0.80; primary motor: 4.80 ± 1  SPECIAL: prefrontal dorsolateral: 6 ± 1; primary motor: 6 ± 1.10 |
| Wijtenburg et al., 2019 | PRESS: Scan 1: 5.40 ± 0.70; Scan 2: 5.30 ± 0.40  MEGA-PRESS: ≤20  PR-STEAM: Scan 1: 4.90 ± 0.30; Scan 2: 4.90 ± 0.30  SPECIAL: Scan 1: 5.20 ± 0.60; Scan 2: 5.30 ± 0.80% | n.p. | n.p. |
| Prisciandaro et al., 2020 | n.p. | n.p. | HERMES: Scan 1: 8.29 ± 0.49; Scan 2: 8.29 ± 0.38  MEGA-PRESS: Scan 1: 7.98 ± 0.51; Scan 2: 8.10 ± 0.69 |

**Supplementary Table S1. Spectral quality of the studies assessing the reproducibility of GSH measurement**

The measures of spectral quality were assessed using SNR, linewidth and CRLB. The method used in each paper to calculate SNR is reported in parentheses below the SNR value, since different definitions produce SNR estimates in different ranges. “LCModel S/N” provided by the LCModel software is defined as the maximum in the spectrum-minus-baseline over the analysis window (which will be for NAA in healthy brain) divided by twice the root-mean-square of the residuals. Abbreviation: CRLB, Cramér-Rao Lower Bound; GSH, glutathione; Hz, hertz; JDE semi-LASER, J-Difference Editing semi-Localized by Adiabatic SElective Refocusing sequence; MEGA-PRESS, MEshcher-GArwood-Point RESolved Spectroscopy; n.p., not provided; PRESS, Point RESolved Spectroscopy; PR-STEAM, Phase Rotation STimulated Echo Acquisition Mode; SPECIAL, SPin Echo full Intensity Acquired Localized; STEAM, STimulated Echo Acquisition Mode.

| **Study** | **PMI** | **Storage temperature** |
| --- | --- | --- |
| Tong et al., 2016 | 3 to 27h | -80°C |
| Venkateshappa et al., 2012 | 4 to 22h | -80°C |
| Harish et al., 2012 | 2.5 to 22h | -80°C |

**Supplementary Table S2. Brain autopsy analysis parameters**

Abbreviation: h, hours; PMI, post-mortem interval; °C, degree Celsius.

| **Authors** | **Blood** | **Plasma** | **Serum** | **Delay until centrifugation** | **Centrifugation time** | **Centrifugation temperature** | **Temperature of storage until analysis** |
| --- | --- | --- | --- | --- | --- | --- | --- |
| Michelet et al., 1995 | Venous | X |  | 2 to 22 min at RT | 10 min | 4°C | -80°C |
| Yang et al., 1995 | Venous | X |  | Immediately | 20 min | 4°C | -20°C (or analysed immediately) |
| Paolisso et al., 1998 | Venous | X |  | Immediately | n.p. | 4°C | -20°C |
| Samiec et al., 1998 | Venous | X |  | Immediately | 30 sec | n.p. | -70°C |
| Hernanz et al., 2000 | Venous | X |  | Immediately | n.p. | 4°C | -70°C |
| Houze et al., 2001 | Venous | X |  | Immediately | 5 min | n.p. | -80°C |
| Jones et al., 2002 | Venous | X |  | Immediately | n.p. | n.p. | -80°C |
| Rea et al., 2004 | Venous | X |  | n.p. | n.p. | n.p. | -7°C |
| Chillemi et al., 2005 | Venous | X |  | Immediately | 10 min | 4°C | -80°C |
| Giustarini et al., 2006 | Venous | X |  | Immediately | 15 sec | 4°C | 0°C |
| Maciejczyk et al., 2019 | Venous | X |  | n.p. | 10 min | 4°C | -80°C |
| Kretzschmar et al., 1991 | Ear | X |  | n.p. | 5 min | n.p. | On ice (3 hours max) |
| Nuttall et al., 1998 | Venous | X |  | Immediately | 15 min | 4°C | -80°C |
| Andriollo-Sanchez et al., 2005 | Venous | X |  | n.p. | 15 min | 4°C | -80°C |
| Pardo-Andreu et al., 2006 | Venous |  | X | n.p. | n.p. | n.p. | -20°C |
| Pérez et al., 2020 | Venous |  | X | n.p. | n.p. | n.p. | -80°C |

**Supplementary Table S3: Blood analyses parameters**

Centrifugation was done at a cold temperature in all studies providing centrifugation temperature (N=9), with -80°C being the most frequently used storage temperature. Abbreviation: min, minute; n.p., not provided; RT, room temperature; °C, degree Celsius.

| **Title** | **Population** | **Treatment** | | **GSH-associated compounds measurement** | |
| --- | --- | --- | --- | --- | --- |
|  |  | **Category** | **Type** | **Compound** | **Measurement** |
| Effects of a Recreational Team Handball-based Programme on Health and Physical Fitness of Middle-aged and Older Men ((H4HM)) | Healthy aging | Physical exercise | Handball | GR  GPx | Blood plasma |
| The Safety and Efficacy Study of RiaGev in Healthy Adults | Healthy aging | Dietary supplement | RiaGev™: Bioenergy Ribose® and vitamin B3 | tGSH | Blood serum |
| Dairy Intake and Brain Health in Aging | Healthy aging | Dietary supplement | Dairy food | GSH | Brain (MRS) |
| Effects of Blueberry Juice Consumption on Cognitive Function in Healthy Older People | Healthy aging | Dietary supplement | Blueberry concentrate | GSH | Brain |
| Study of the Effects of Cerefolin NAC on Inflammation Blood Markers in Older Individuals with Memory Complaints | Subjective memory loss | Dietary supplement | NAC | GSH | Blood plasma |
| Nutritional Intervention with the Dietary Supplement, Immunocal® in MCI Patients: Promotion of Brain Health | MCI | Dietary supplement | Immunocal: cysteine-rich whey protein isolate | GSH | Blood plasma;  Brain (MRS) |
| The Effect of Consumption of Almonds and Snack Mix Daily for 6 Months on Cognitive Function in Older Adults | MCI | Dietary supplement | Almonds | GPx  GR | Blood serum |
| Brazil Nuts Effects on Selenium Status and Cognitive Performance | MCI | Dietary supplement | Brazil nuts | GPx | Blood plasma;  Erythrocytes |
| Improving Effects of Fish Oil Combined with Pine Bark Extract on Cognitive Decline | MCI | Dietary supplement | Fish oil and pine extract | GSH/GSSG | Blood |
| Anthocyanins as Dementia Prevention? (ACID) | MCI | Dietary supplement | Anthocyanins | GSH | Blood |
| Chocolate and Physical Exercise to Reduce Malnutrition in Pre-dementia Aged People (Choko-AGE) | MCI;  Mild dementia | Physical exercise | Aerobic and strength training exercises | GSH | Biopsy muscle tissue |
|  |  | Dietary supplement | Protein-rich diet;  Dark chocolate with total polyphenol and vitamin E |  |  |
| Effects of Nicotinamide Riboside on Bioenergetics and Oxidative Stress in Mild Cognitive Impairment/Alzheimer's Dementia | MCI;  Mild AD | Drug | Nicotinamide riboside | GSH | Brain |
| Insulin-Sensitizing Anti-Inflammatory Small Molecule for Investigative Treatment of Dementia | MCI;  Mild AD | Drug | NE3107 | GSH | Brain (MRS) |
| Glutathione, Brain Metabolism, and Inflammation in Alzheimer's Disease | AD | Dietary supplement | Glycine;  NAC | GSH not assessed | |
| Intranasal Insulin and Glutathione as an Add-On Therapy in Parkinson's Disease (NOSE-PD) | PD | Drug | GSH (intranasal) | GSH not assessed | |
| Glutathione (GSH) In the Treatment of Parkinson's Disease | PD | Drug | GSH (intravenous) | GSH not assessed | |
| CNS Uptake of Intranasal Glutathione | PD | Drug | GSH (intranasal) | GSH | Red blood cells;  Brain (MRS) |
| Effect of Aerobic Training on Oxidative Stress Markers in Patients with Parkinson's Disease | PD | Physical exercise | Physiotherapy;  Rehabilitation program | GSH | Not specified |
| N-Acetylcysteine for Neuroprotection in Parkinson's Disease (NAC for PD) | PD | Drug | NAC | GSH | Brain (MRS) |
| Repeated-Dose Oral N-acetylcysteine for the Treatment of Parkinson's Disease | PD | Drug | NAC | GSH | Blood plasma;  Red blood cells;  Brain (MRS) |
| Effects of Yoga on Parkinson's Disease (HYPD) | PD | Physical exercise | Hatha yoga | GSH  GSH/GSSG | Blood |
| Vitamin B6, B12, Folic Acid and Exercise in Parkinson's Disease | PD | Dietary supplement | Vitamin B6;  Vitamin B12;  Folic acid | GSH  GSH/GSSG | Blood plasma |
|  |  | Physical exercise | n.p. |  |  |
| Safety and Biomarker Study of PTC-589 in Participants with Parkinson's Disease | PD | Drug | PTC-589 | GSH | Blood plasma;  Urine;  CSF |
| Effect of Undenatured Cysteine-Rich Whey Protein Isolate (HMS 90®) in Patients with Parkinson's Disease | PD | Dietary supplement | Whey protein;  Soy protein | GSH | Blood plasma |
| Effects of Diffused Ylang-Ylang Essential Oil Amongst Older Persons with Dementia | Dementia or BPSD | Aromatherapy | Ylang-Ylang essential oil | GSH | Blood |

**Supplementary Table S4: Clinical trials information**

Note: we have removed clinical trials (N=3) only looking at red blood cells or erythrocytes. Abbreviation: AD, Alzheimer’s disease; BPSD, behavioral and psychological symptoms of dementia; CSF, cerebrospinal fluid; GPx, glutathione peroxidase; GR, glutathione reductase; GSH, glutathione; GSSG, glutathione disulfide; MCI, mild cognitive impairment; MRS, magnetic resonance spectroscopy; NAC, N-acetylcysteine; n.p., not provided; PD, Parkinson’s disease; tGSH, total glutathione.
