# Supplementary material for: Changes in levels of the antioxidant glutathione in brain and blood across the age span of healthy adults: A systematic review
Source: Neuroimage Clin. 2023 Aug 26;40:103503. doi: 10.1016/j.nicl.2023.103503 (PMC10520675; doi:10.1016/j.nicl.2023.103503)
Supplement: Supplementary data 2 [file mmc2.docx]

| **Study** | **GSH CRLB (%)** | **SNR**  **(Method)** | **Linewidth (Hz)** |
| --- | --- | --- | --- |
| van de Bank et al., 2015 | Corona radiata: 12.60  Posterior cingulate: 13.20 | n.p. | n.p. |
| Lally et al., 2016 | Day 1/Scan 1: 6.14 ± 2.20  Day 1/Scan 2: 6.13 ± 1.38  Day 2/Scan 1: 5.83 ± 2.25  Day 2/Scan 2: 5.35 ± 0.82 | n.p. | 12.14 ± 1.48 |
| Prinsen et al., 2017 | STEAM: 5.70  JDE semi-LASER: 3 | n.p. | STEAM: 10  JDE semi-LASER: 12.2 ± 1.2 |
| Gonen et al., 2020 | 5.0 ± 0.8 | n.p. | n.p. |
| Lim & Xin, 2022 | MEGA-sPECIAL: 19.50  sSPECIAL: 7 | sSPECIAL: motor cortex: Scan 1: 548 ± 51, Scan 2: 587 ± 86; Medial prefrontal: Scan 1: 486 ± 59, Scan 2: 504 ± 32  MEGA-sSPECIAL-edit-off: motor cortex: Scan 1: 235 ± 60, Scan 2: 236 ± 53; Medial prefrontal: Scan 1: 257 ± 34, Scan 2: 272 ± 28  (Height of the NAA peak at 2.02 ppm divided by the standard deviation of the noise between 9.5 and 10 ppm) | sSPECIAL: motor cortex: Scan 1: 12 ± 0.80, Scan 2: 11.90 ± 0.40; Medial prefrontal: Scan 1: 12.60 ± 1.10, Scan 2: 13.70 ± 2.10  MEGA-sSPECIAL: motor cortex: Scan 1: 12.40 ± 0.90, Scan 2: 12.10 ± 1; Medial prefrontal: Scan 1: 12.70 ± 0.70, Scan 2: 12.50 ± 1.20 |
| Reid et al., 2022 | Scan 1: 4.30  Scan 2: 4.70 | Scan 1: 58.80 ± 7.50  Scan 2: 58.30 ± 12.60  (LCModel S/N) | Scan 1: 15.60 ± 2.60  Scan 2: 15.30 ± 1.90 |
| Terpstra et al., 2005 | 14 | High (value not provided) | 6-11 (average: 8) |
| Wijtenburg et al., 2014 | Anterior cingulate: Scan 1: 6.40; Scan 2: 6.40  Posterior cingulate: Scan 1: 7.60; Scan 2: 8.20 | Anterior cingulate: Scan 1: 36.60; Scan 2: 33.50  Posterior cingulate: Scan 1: 32.30; Scan 2: 31.10  (LCModel S/N) | Anterior cingulate: Scan 1: 0.02; Scan 2: 0.03  Posterior cingulate: Scan 1: 0.02; Scan 2: 0.02 |
| Bednařík et al., 2015 | <20 | 53.60 ± 8.10  (Amplitude of the NAA resonance at 2.02 ppm, divided by the root-mean-square of the noise on the summed spectrum) | 9 |
| Schubert et al., 2017 | 24 ± 11 | 21 ± 5  (LCModel S/N) | 8 |
| Dhamala et al., 2019 | n.p. | MEGA-PRESS: prefrontal dorsolateral: 337 ± 57; primary motor: 290 ± 56  SPECIAL: prefrontal dorsolateral: 693 ± 80; primary motor: 607 ± 87  (Amplitude of the NAA peak, divided by the standard deviation of the noise within spectral windows [−1.4, −0.4] ppm and [9, 10] ppm) | MEGA-PRESS: prefrontal dorsolateral: 4.40 ± 0.80; primary motor: 4.80 ± 1  SPECIAL: prefrontal dorsolateral: 6 ± 1; primary motor: 6 ± 1.10 |
| Wijtenburg et al., 2019 | PRESS: Scan 1: 5.40 ± 0.70; Scan 2: 5.30 ± 0.40  MEGA-PRESS: ≤20  PR-STEAM: Scan 1: 4.90 ± 0.30; Scan 2: 4.90 ± 0.30  SPECIAL: Scan 1: 5.20 ± 0.60; Scan 2: 5.30 ± 0.80% | n.p. | n.p. |
| Prisciandaro et al., 2020 | n.p. | n.p. | HERMES: Scan 1: 8.29 ± 0.49; Scan 2: 8.29 ± 0.38  MEGA-PRESS: Scan 1: 7.98 ± 0.51; Scan 2: 8.10 ± 0.69 |

**Supplementary Table S1. Spectral quality of the studies assessing the reproducibility of GSH measurement**

The measures of spectral quality were assessed using SNR, linewidth and CRLB. The method used in each paper to calculate SNR is reported in parentheses below the SNR value, since different definitions produce SNR estimates in different ranges. “LCModel S/N” provided by the LCModel software is defined as the maximum in the spectrum-minus-baseline over the analysis window (which will be for NAA in healthy brain) divided by twice the root-mean-square of the residuals. Abbreviation: CRLB, Cramér-Rao Lower Bound; GSH, glutathione; Hz, hertz; JDE semi-LASER, J-Difference Editing semi-Localized by Adiabatic SElective Refocusing sequence; MEGA-PRESS, MEshcher-GArwood-Point RESolved Spectroscopy; n.p., not provided; PRESS, Point RESolved Spectroscopy; PR-STEAM, Phase Rotation STimulated Echo Acquisition Mode; SPECIAL, SPin Echo full Intensity Acquired Localized; STEAM, STimulated Echo Acquisition Mode.
