# Supplementary material for: Changes in levels of the antioxidant glutathione in brain and blood across the age span of healthy adults: A systematic review
Source: Neuroimage Clin. 2023 Aug 26;40:103503. doi: 10.1016/j.nicl.2023.103503 (PMC10520675; doi:10.1016/j.nicl.2023.103503)
Supplement: Supplementary data 3 [file mmc3.docx]

| **Study** | **PMI** | **Storage temperature** |
| --- | --- | --- |
| Tong et al., 2016 | 3 to 27h | -80°C |
| Venkateshappa et al., 2012 | 4 to 22h | -80°C |
| Harish et al., 2012 | 2.5 to 22h | -80°C |

**Supplementary Table S2. Brain autopsy analysis parameters**

Abbreviation: h, hours; PMI, post mortem interval;°C, degree Celsius.
