# Supplementary material for: Changes in levels of the antioxidant glutathione in brain and blood across the age span of healthy adults: A systematic review
Source: Neuroimage Clin. 2023 Aug 26;40:103503. doi: 10.1016/j.nicl.2023.103503 (PMC10520675; doi:10.1016/j.nicl.2023.103503)
Supplement: Supplementary data 4 [file mmc4.docx]

| **Authors** | **Blood** | **Plasma** | **Serum** | **Delay until centrifugation** | **Centrifugation time** | **Centrifugation temperature** | **Temperature of storage until analysis** |
| --- | --- | --- | --- | --- | --- | --- | --- |
| Michelet et al., 1995 | Venous | X |  | 2 to 22 min at RT | 10 min | 4°C | -80°C |
| Yang et al., 1995 | Venous | X |  | Immediately | 20 min | 4°C | -20°C (or analysed immediately) |
| Paolisso et al., 1998 | Venous | X |  | Immediately | n.p. | 4°C | -20°C |
| Samiec et al., 1998 | Venous | X |  | Immediately | 30 sec | n.p. | -70°C |
| Hernanz et al., 2000 | Venous | X |  | Immediately | n.p. | 4°C | -70°C |
| Houze et al., 2001 | Venous | X |  | Immediately | 5 min | n.p. | -80°C |
| Jones et al., 2002 | Venous | X |  | Immediately | n.p. | n.p. | -80°C |
| Rea et al., 2004 | Venous | X |  | n.p. | n.p. | n.p. | -7°C |
| Chillemi et al., 2005 | Venous | X |  | Immediately | 10 min | 4°C | -80°C |
| Giustarini et al., 2006 | Venous | X |  | Immediately | 15 sec | 4°C | 0°C |
| Maciejczyk et al., 2019 | Venous | X |  | n.p. | 10 min | 4°C | -80°C |
| Kretzschmar et al., 1991 | Ear | X |  | n.p. | 5 min | n.p. | On ice (3 hours max) |
| Nuttall et al., 1998 | Venous | X |  | Immediately | 15 min | 4°C | -80°C |
| Andriollo-Sanchez et al., 2005 | Venous | X |  | n.p. | 15 min | 4°C | -80°C |
| Pardo-Andreu et al., 2006 | Venous |  | X | n.p. | n.p. | n.p. | -20°C |
| Pérez et al., 2020 | Venous |  | X | n.p. | n.p. | n.p. | -80°C |

**Supplementary Table S3: Blood analyses parameters**

Centrifugation was done at a cold temperature in all studies providing centrifugation temperature (N=9), with -80°C being the most frequently used storage temperature. Abbreviation: min, minute; n.p., not provided; RT, room temperature; °C, degree Celsius.
