# Supplementary material for: Changes in levels of the antioxidant glutathione in brain and blood across the age span of healthy adults: A systematic review
Source: Neuroimage Clin. 2023 Aug 26;40:103503. doi: 10.1016/j.nicl.2023.103503 (PMC10520675; doi:10.1016/j.nicl.2023.103503)
Supplement: Supplementary data 5 [file mmc5.docx]

| **Title** | **Population** | **Treatment** | | **GSH-associated compounds measurement** | |
| --- | --- | --- | --- | --- | --- |
|  |  | **Category** | **Type** | **Compound** | **Measurement** |
| Effects of a Recreational Team Handball-based Programme on Health and Physical Fitness of Middle-aged and Older Men ((H4HM)) | Healthy aging | Physical exercise | Handball | GR  GPx | Blood plasma |
| The Safety and Efficacy Study of RiaGev in Healthy Adults | Healthy aging | Dietary supplement | RiaGev™: Bioenergy Ribose® and vitamin B3 | tGSH | Blood serum |
| Dairy Intake and Brain Health in Aging | Healthy aging | Dietary supplement | Dairy food | GSH | Brain (MRS) |
| Effects of Blueberry Juice Consumption on Cognitive Function in Healthy Older People | Healthy aging | Dietary supplement | Blueberry concentrate | GSH | Brain |
| Study of the Effects of Cerefolin NAC on Inflammation Blood Markers in Older Individuals with Memory Complaints | Subjective memory loss | Dietary supplement | NAC | GSH | Blood plasma |
| Nutritional Intervention with the Dietary Supplement, Immunocal® in MCI Patients: Promotion of Brain Health | MCI | Dietary supplement | Immunocal: cysteine-rich whey protein isolate | GSH | Blood plasma;  Brain (MRS) |
| The Effect of Consumption of Almonds and Snack Mix Daily for 6 Months on Cognitive Function in Older Adults | MCI | Dietary supplement | Almonds | GPx  GR | Blood serum |
| Brazil Nuts Effects on Selenium Status and Cognitive Performance | MCI | Dietary supplement | Brazil nuts | GPx | Blood plasma;  Erythrocytes |
| Improving Effects of Fish Oil Combined with Pine Bark Extract on Cognitive Decline | MCI | Dietary supplement | Fish oil and pine extract | GSH/GSSG | Blood |
| Anthocyanins as Dementia Prevention? (ACID) | MCI | Dietary supplement | Anthocyanins | GSH | Blood |
| Chocolate and Physical Exercise to Reduce Malnutrition in Pre-dementia Aged People (Choko-AGE) | MCI;  Mild dementia | Physical exercise | Aerobic and strength training exercises | GSH | Biopsy muscle tissue |
|  |  | Dietary supplement | Protein-rich diet;  Dark chocolate with total polyphenol and vitamin E |  |  |
| Effects of Nicotinamide Riboside on Bioenergetics and Oxidative Stress in Mild Cognitive Impairment/Alzheimer's Dementia | MCI;  Mild AD | Drug | Nicotinamide riboside | GSH | Brain |
| Insulin-Sensitizing Anti-Inflammatory Small Molecule for Investigative Treatment of Dementia | MCI;  Mild AD | Drug | NE3107 | GSH | Brain (MRS) |
| Glutathione, Brain Metabolism, and Inflammation in Alzheimer's Disease | AD | Dietary supplement | Glycine;  NAC | GSH not assessed | |
| Intranasal Insulin and Glutathione as an Add-On Therapy in Parkinson's Disease (NOSE-PD) | PD | Drug | GSH (intranasal) | GSH not assessed | |
| Glutathione (GSH) In the Treatment of Parkinson's Disease | PD | Drug | GSH (intravenous) | GSH not assessed | |
| CNS Uptake of Intranasal Glutathione | PD | Drug | GSH (intranasal) | GSH | Red blood cells;  Brain (MRS) |
| Effect of Aerobic Training on Oxidative Stress Markers in Patients with Parkinson's Disease | PD | Physical exercise | Physiotherapy;  Rehabilitation program | GSH | Not specified |
| N-Acetylcysteine for Neuroprotection in Parkinson's Disease (NAC for PD) | PD | Drug | NAC | GSH | Brain (MRS) |
| Repeated-Dose Oral N-acetylcysteine for the Treatment of Parkinson's Disease | PD | Drug | NAC | GSH | Blood plasma;  Red blood cells;  Brain (MRS) |
| Effects of Yoga on Parkinson's Disease (HYPD) | PD | Physical exercise | Hatha yoga | GSH  GSH/GSSG | Blood |
| Vitamin B6, B12, Folic Acid and Exercise in Parkinson's Disease | PD | Dietary supplement | Vitamin B6;  Vitamin B12;  Folic acid | GSH  GSH/GSSG | Blood plasma |
|  |  | Physical exercise | n.p. |  |  |
| Safety and Biomarker Study of PTC-589 in Participants with Parkinson's Disease | PD | Drug | PTC-589 | GSH | Blood plasma;  Urine;  CSF |
| Effect of Undenatured Cysteine-Rich Whey Protein Isolate (HMS 90®) in Patients with Parkinson's Disease | PD | Dietary supplement | Whey protein;  Soy protein | GSH | Blood plasma |
| Effects of Diffused Ylang-Ylang Essential Oil Amongst Older Persons with Dementia | Dementia or BPSD | Aromatherapy | Ylang-Ylang essential oil | GSH | Blood |

**Supplementary Table S4: Clinical trials information**

Note: we have removed clinical trials (N=3) only looking at red blood cells or erythrocytes. Abbreviation: AD, Alzheimer’s disease; BPSD, behavioral and psychological symptoms of dementia; CSF, cerebrospinal fluid; GPx, glutathione peroxidase; GR, glutathione reductase; GSH, glutathione; GSSG, glutathione disulfide; MCI, mild cognitive impairment; MRS, magnetic resonance spectroscopy; NAC, N-acetylcysteine; n.p., not provided; PD, Parkinson’s disease; tGSH, total glutathione.
